# Digital circadian and sleep health in individual hospital shift workers: A cross sectional telemonitoring study

**DOI:** 10.1016/j.ebiom.2022.104121

**Published:** 2022-06-27

**Authors:** Yiyuan Zhang, Emilie Cordina-Duverger, Sandra Komarzynski, Amal M. Attari, Qi Huang, Guillen Aristizabal, Brice Faraut, Damien Léger, René Adam, Pascal Guénel, Julia A. Brettschneider, Bärbel F. Finkenstädt, Francis Lévi

**Affiliations:** a Department of Statistics, University of Warwick, Coventry, United Kingdom; b Inserm, CESP, Team Exposome and Heredity, University Paris-Saclay, Gustave Roussy, Villejuif, France; c Cancer Chronotherapy Team, Cancer Research Centre, Division of Biomedical Sciences, Warwick Medical School, University of Warwick, Coventry, United Kingdom; d UPR “Chronothérapie, Cancers, et Transplantation”, Faculté de Médecine, Université Paris-Saclay, Villejuif, France; e Cap Gemini, Velizy Villacoublay, France; f Université de Paris, VIFASOM (EA 7330 Vigilance Fatigue, Sommeil et Santé Publique), Paris, France; g Assistance Publique-Hôpitaux de Paris, APHP-Centre Université de Paris, Hôtel Dieu, Centre du Sommeil et de La Vigilance, Paris, France; h Hepato-Biliary Center, Paul Brousse Hospital, Assistance Publique-Hôpitaux de Paris, Villejuif, France; i Department of Medical Oncology, Paul Brousse Hospital, Assistance Publique-Hôpitaux de Paris, Villejuif, France

**Keywords:** Circadian rhythm, Rest-activity, Sleep, Body temperature, Shift work, Telemonitoring

## Abstract

**Background:**

Telemonitoring of circadian and sleep cycles could identify shift workers at increased risk of poor health, including cancer and cardiovascular diseases, thus supporting personalized prevention.

**Methods:**

The Circadiem cross-sectional study aimed at determining early warning signals of risk of health alteration in hospital nightshifters (NS) versus dayshifters (DS, alternating morning and afternoon shifts). Circadian rhythmicity in activity, sleep, and temperature was telemonitored on work and free days for one week. Participants wore a bluetooth low energy thoracic accelerometry and temperature sensor that was wirelessly connected to a GPRS gateway and a health data hub server. Hidden Markov modelling of activity quantified Rhythm Index, rest quality (probability, p1-1, of remaining at rest), and rest duration. Spectral analyses determined periods in body surface temperature and accelerometry. Parameters were compared and predictors of circadian and sleep disruption were identified by multivariate analyses using information criteria-based model selection. Clusters of individual shift work response profiles were recognized.

**Findings:**

Of 140 per-protocol participants (133 females), there were 63 NS and 77 DS. Both groups had similar median rest amount, yet NS had significantly worse median rest-activity Rhythm Index (0·38 [IQR, 0·29-0·47] *vs.* 0·69 [0·60-0·77], p<0·0001) and rest quality p1-1 (0·94 [0·94-0·95] vs 0·96 [0·94-0·97], p<0·0001) over the whole study week. Only 48% of the NS displayed a circadian period in temperature, as compared to 70% of the DS (p*=*0·026). Poor p1-1 was associated with nightshift work on both work (p<0·0001) and free days (p*=*0·0098). The number of years of past night work exposure predicted poor rest-activity Rhythm Index jointly with shift type, age and chronotype on workdays (p= 0·0074), and singly on free days (p=0·0005).

**Interpretation:**

A dedicated analysis toolbox of streamed data from a wearable device identified circadian and sleep rhythm markers, that constitute surrogate candidate endpoints of poor health risk in shift-workers.

**Funding:**

French Agency for Food, Environmental and Occupational Health & Safety (EST-2014/1/064), University of Warwick, Medical Research Council (United Kingdom, MR/M013170), Cancer Research UK(C53561/A19933).

**Research in context**

**Evidence before this study**

Nightshift work is essential for the activities of our societies, with shift workers representing 15 to 30% of all workers worldwide, despite the fact that it is frequently connected with suffering from circadian disruption and insomnia.^1^ Consistent associations have been found between night work and risks of malignant, cardiovascular and other diseases,^2^, ^3^, ^4^, ^5^, ^6^, ^7^ and have been further highlighted in exhaustive reports by public health organisations, notably the International Agency for Research on Cancer,^1^ the US National Toxicology Program (https://ntp.niehs.nih.gov/ntp/about_ntp/monopeerrvw/2018/october/landraftmonograph20180824.pdf), and the French Agency for Food, Environmental and Occupational Health & Safety (ANSES, https://www.anses.fr/en/system/files/AP2011SA0088RaEN.pdf). In a pooled analysis of five population-based case–control studies, night-work increased the breast cancer risk in premenopausal women working in nightshifts lasting at least 10 hours each (Odds Ratio, OR, 1·36 [95% CI, 1·07-1·74]). Working for three or more nights per week for 10 years or more produced the highest OR (2·55 [1·03-6·20]).^7^ Similarly, a large meta-analysis of 21 studies showed increased risk among shift workers compared to those who did not work in shifts (OR 1·22 [1·09-1·37] for cardiovascular disease and 1·18 [1·06-1·32] for coronary heart disease mortality).^5^ After the first five years of shift work, there was a 7·1% increase in risk of cardiovascular disease events for every additional five years of exposure.^5^

**Added value of this study**

The Circadiem cross-sectional study reveals the imprinting of circadian rhythm and sleep disruptions in association with prolonged night work, a main determinant of cancer and cardiovascular risk. The study was conducted according to STROBE guidelines^8^ in 63 nightshifters working three or more nights of ∼10h each per week, and 77 dayshifters alternating morning and afternoon shifts at a single university hospital in Paris (France). It offers the integrated analysis of shift workers’ rest-activity and body temperature rhythms within a comprehensive statistical framework that takes account of circadian rhythms, combining classical epidemiology with concepts from personalized medicine.

Nightshift work nearly halved the median rest-activity Rhythm Index and suppressed circadian temperature rhythm in 48% of the health professionals. Substantial variability was observed in measures of circadian disruption among night workers. Analysis of individual daily rest profiles resolved the subjects into three clusters. Among nightshifters, a first cluster included those who coped best with night work, showing limited changes in their rest-activity patterns between work and free days. Those in a second cluster suffered from social jet lag on night-work days, but their Rhythm Index fully recovered on free days, whilst their centre time of rest advanced by 7·6 h on average. In contrast, a third cluster of 11 nightshifters (17·5%) was characterised by lowest Rhythm Index on both work and free days, and shortest ultradian cycles during sleep. The nightshifters in this cluster had on average nearly twice as much past exposure to night work as subjects in the other two clusters. Indeed, multivariate analysis revealed that the number of years of past night work predicted poor rest-activity Rhythm Index, both on free days (p=0·0005) and – together with shift type, age, and chronotype – on workdays (p=0·0074).

**Implications of all the available evidence**

Chest rest-activity Rhythm Index, along with chest surface temperature rhythm patterns are potential surrogates for cancer and cardiovascular disease risk increase related to night work exposure. We show that these rhythms can be easily telemonitored for one week in active healthcare professionals. The analysis of ultradian cyclicity in physiological parameters during sleep, measured non-intrusively by wearable devices, is a promising direction relevant to sleep research and sleep disorders at large. Our study provides an effective pipeline for the integrated statistical analysis of multidimensional telemonitoring data combining multivariate regression with data-driven model selection, machine learning tools, and spectral analysis. Most importantly, the critical quantitative parameters that estimate circadian and sleep health can be automatically computed and reported, as already implemented in an ongoing cancer trial (https://www.clinicaltrials.gov/ct2/show/NCT04263948).

Our results stress the need for further longitudinal telemonitoring epidemiologic studies to firmly establish chest rest-activity and temperature rhythm as early warning signals and measurable surrogates of disease risk at subclinical stages. Such indicators could be used to design medically sound interventions for adapting work schedules to individual workers in a new era of personalized prevention medicine.

Alt-text: Unlabelled box

## Introduction

Shift work involves about 20% of the working population worldwide, including healthcare professionals.^1^^,^^2^ Epidemiological studies and international health agency reports have revealed consistent associations between rotating or night shift work and increased risk of sleep disorders, daily sleepiness, work accidents, metabolic syndrome, type 2 diabetes, obesity, dyslipidemia, coronary ischemic disease, high blood pressure, and cancer.^1^, ^2^, ^3^, ^4^, ^5^, ^6^ The increase in disease risk was found to be consistently highest for both cancer and cardiovascular diseases among those exposed to night shift work for 5 or more years.^1^^,^^5^^,^^7^

The detection of early warning signals of health alterations based on circadian and sleep cycles disorders could later drive individual data-based preventive interventions, while monitoring their efficacy in shift workers. Thus, poor health can result from circadian and sleep disruption, due to frequent changes in the environmental light and other cycles which synchronize the circadian timing system (CTS).^1^^,^^9^ In health, the CTS rhythmically regulates behaviour and physiology, as well as hormonal secretions, metabolism, proliferation, DNA repair, and apoptosis over the 24-hours.^9^^,^^10^ The CTS represents a complex network of molecular clocks that reside within each cell and are coordinated by a central pacemaker, the suprachiasmatic nuclei (SCN) in the hypothalamus.^10^ These nuclei generate the endogenous circadian rhythms in rest-activity and body temperature, which have been proposed as CTS biomarkers^10^ and are used here for such purpose. Our current understanding of circadian rhythms as a network of molecular clocks and their relevance for human health has quickly progressed^11^^,^^12^ and it is now imminent that they become integrated into medical decision processes.^13^, ^14^, ^15^

Shiftwork-induced CTS disruption is experimentally-modelled with chronic jet lag exposure. Repeat alterations of light-dark cycles in rodents suppresses, dampens and phase shifts the physiological 24-hour rhythms in rest-activity, body temperature, glucocorticoids, hormonal secretions, and clock genes expressions in many tissues,^9^ resulting in a drastic increase in incidence or a severe worsening for cardiac pathologies^16^^,^^17^; atherosclerosis^18^; inflammatory liver or intestinal diseases^19^, ^20^, ^21^; and breast, lung, liver and other cancers.^22^, ^23^, ^24^, ^25^, ^26^, ^27^, ^28^

The current study addresses unmet scientific, technological, and algorithmic needs through physiological telemonitoring during real life, thus permitting the implementation of personalized risk monitoring strategies in individual shift workers. Indeed, circadian rhythms can vary largely between people, with phase differences being partly estimated with the help of morningness-eveningness or chronotype questionnaires.^29^ The CTS response dynamics however can only be tracked through the longitudinal telemonitoring of CTS biomarkers. Prior studies using wrist actimetry in health professionals undergoing rotating or night shift work^24^^,^^25^ have not attempted to identify distinct individual responses to the same work schedule. Using the first mobile multidimensional e-Health platform PiCADo, we uncovered large differences in circadian coordination and sleep features among 88 human subjects and 37 cancer patients, whose rest-activity and body temperature rhythms were telemonitored during their daily life for up to 4 weeks.^30^, ^31^, ^32^

As the circadian rhythm coordinates sleep homeostasis with environmental cues,^11^ a deterioration of an individual's sleep/wake pattern is also marking circadian disruption and associated increased risk of disease. Here, we hypothesized that an increased risk could be detected in individual hospital health care professionals through the response of telemonitored CTS biomarkers rhythms in physical activity (PA) and chest surface temperature (Chesttemp) to night shift work exposure, using the PiCADo platform.^30^

Toward this goal, we first computed four summary statistics of PA rhythms that are informative of both the quality and quantity of rest, and their regular alternations over the 24 hours for each participant. We then developed an original multidimensional toolbox combining multiple statistical methods in order to appraise responses of circadian and sleep cycles to work schedules both in groups of workers and in individuals, and to identify potential determinants of increased risks for health.

## Methods

### Ethics

The Circadiem study aimed at the identification of circadian and sleep disorders as a function of shift work schedule in nurses and other health professionals working in the same public hospital. The study was sponsored by the Institut National de la Santé et de la Recherche Médicale (File #C15-66, INSERM, Paris, France). The protocol was approved by the “Comité consultatif sur le traitement de l'information en matière de recherche dans le domaine de la santé” (File #16.153, approval granted on 23/03/2016), by the Ethical Committee of Ile-de France 1 (on 09/05/2016), and by the “Commission Nationale Informatique et Libertés” (January 2017). Recruitment was advertised through protocol presentations by the study coordinator at meetings that took place in each service (i.e. surgery, oncology, geriatrics, psychiatry, biochemistry, etc.) within Paul-Brousse Hospital, Villejuif, France. This hospital is part of the Assistance Publique - Hôpitaux de Paris (AP-HP), the largest French public hospital institution.

Potential participants were instructed in detail about their expected tasks throughout the 6 to 11-day study period involving both work and free days and were included in the study after signing an informed consent form.

### Study design and participants

Participants in the cross-sectional study Circadiem were non-pregnant female or male health nurses or nurse assistants, aged 25 to 65 years, and working on permanent night or day shifts for a minimum of 5 years. Immediately following their inclusion into the study, they were asked to fill out a detailed sleep questionnaire, the French translation of the morningness-eveningness questionnaire^29^^,^^33^ and questionnaires about lifestyle, including alcohol consumption, smoking, and coffee intake, as well as about socio-familial environment, including cohabitation and the number of children at home. Sleep debt^34^ defined as self-reported ideal total sleep time minus total sleep time (both values were in the sleep questionnaire) was calculated. Participants were categorized as day-shifters (DS) who worked on morning shifts (7:00-14:00), alternating with afternoon shifts (14:00-21:00), or night shifters (21:00-7:00). Actual work and rest schedules were captured from daily self-reported diaries. A “workday” was defined as 24-hour timespan starting at 00:00 for DS and at the time when night shift work began (20:00/21:00) for NS, the remaining timespan defined as “free day”. During a 7-day study period, they were telemonitored by an anterior chest-worn thoracic sensor (Movisens, Karlsruhe, Germany) as part of the PiCaDo mobile e-Health platform (supplementary Figure S1).^30^ Per-minute accelerations measuring physical activity (PA) and chest surface temperature (Chesttemp) were measured and teletransmitted daily. Using PA data collected by wearable sensors to monitor circadian rhythm and sleep pattern has been adopted as a study protocol outside laboratory settings.^35^, ^36^, ^37^ Details on the actual activity such as walking, running, or sitting are not visible in the current telemetric setting. Despite the availability of 3D-orientation from sensor data in 1-minute resolution, pilot studies showed that their additional use in fitting the the hidden Markov models (HMMs) provided no significant improvement in the prediction of the HMM states as compared to using acceleration measurements data only.

### Statistics

The current study aimed to identify those individual health professionals who experienced circadian disruption on a standard work schedule (i.e. DS or NS only). In order to be able to assess recovery from circadian disruption we selected the DS and NS who had at least 1 workday and at least 1 free day during the monitoring period. Our goal was to include approximately similar numbers of DS and NS. We targeted a samples size of 50-70 people per shift schedule to be telemonitored for a week, based on the demonstration of statistically significant differences in circadian rhythms in rest-activity and/or sleep as a function of day or night work schedule in earlier published reports^38^ and more recent ones.^39^, ^40^, ^41^ We further found such a sample size estimate would confer adequate power to a comparison of the Rhythm Index (RI) and p1-1 between dayshifters and nightshifters using respective standard deviations of RI and p1-1 of 0·14 and 0·03 as found in a prior study in cancer patients^32^ (Lévi, Finkenstadt and Brettschneider, unpublished data). Assuming that the SD values were good enough approximations for other populations, a sample size of 64 per group would allow the detection of a difference in means of 0·7 in RI and 0·015 in p1-1 (each corresponding to Cohen's d of 0·5, hence a medium effect size) at 95% confidence with 80% power.

The following methods were applied for the determination of circadian and sleep parameters in each individual participant and to the identification of groups of participants with different responses to the day or night shift schedules.

#### Hidden Markov model

This model was applied for the determination of circadian rest-activity and sleep parameters. In the hidden Markov model (HMM), the PA data are modelled as a sequence of realizations of a set of probability distributions, called emissions, one for each state of an underlying Markov chain assumed to be unobserved (or hidden). The HMM is parameterised by its matrix of transition probabilities, i.e. the probabilities of the Markov chain going to state i when it was previously in state j, the emission densities and the initial state distribution. Assuming Gaussian emissions the model was fitted individually to the square root of 5-min average PA data to classify the activity of each individual into three possible states: inactive (rest), moderately active, or highly active. The *harmonic* HMM (HHMM) proposed for accelerometry data^31^ assumes that the transition probabilities are influenced by a circadian oscillator. Furthermore, the *two-oscillator HHMM* (2-HHMM), allows for this circadian oscillator to vary between work and free days. Based on the HMM four *circadian parameters*^31^ were computed for each subject, both over the entire study session and separately for work and free days (see the List of parameters and indicators).

List of parameters and indicators

- **p1-1** represents the probability of staying in the rest state having been in the rest state in the previous period. The larger the value of p1-1 the less interruptions occurred and the better the quality of rest.
- **rest profile**: the curve of the probability of the rest state over the 24-hours.
- **rest amount**: the area under the rest profile, ranging from 0h to 24h.
- **centre time of rest**: the gravity centre of the rest amount, ranging from 0:00 to 23:59.
- **rhythm index (RI)**: given the individual's rest amount and centre time of rest, the RI is an index that takes a value of 1 if the subject has a perfectly regular daily recurrence of rest and is in rest state with probability 1, and a value of 0 in the absence of any circadian cycle in rest, thus measuring regularity and quality of rest.

All parameters and indicators are estimated from the fitted HHMM or 2-HHMM (see Supplementary Material, SM-2).

#### Cluster analysis

Health professionals allocated to the same shift type were further classified into subgroups that shared common rest patterns by applying cluster analysis to the average daily ‘rest profiles’ (see the List of parameters and indicators). The rest profiles for work and free days were concatenated into a vector which thus contained 576 elements for each participant, i.e. 288 entries for each rest profile evaluated at 5-minute intervals from 20:00 to 19:59 with data extracted from the averages of either just work or just free days.

Cluster analysis provides an exploratory method to extract the most common patterns by identifying subgroups of subjects with maximal similarity within subgroups and maximal difference between subgroups. It incorporates three tasks: the selection of a distance metric that addresses the differences in the observed profiles between subjects, the choice of a clustering algorithm, and the selection of the number of clusters. Two common distance measures, namely the Euclidean and the Pearson correlation distance, were considered. Agglomerative clustering with average linkage, complete linkage and Ward's method,^42^ and partitional clustering including K-means and K-mediods were first tested on whether they managed to retrospectively categorise the participants into the (known) DS and NS groups. Agglomerative clustering with Ward's method based on the Euclidean distance had the highest correct classification rate and was hence our chosen clustering method. Note that Ward's method determines the distance between two clusters and is based on the increases of the within-cluster sum of squares$\;W_{k}$ that results from merging the two clusters $W_{k}=\sum_{m=1}^{k}\frac{1}{2n_{m}}\sum_{i,j\in C_{m}}d\left(x_{i},x_{j}\right)$, where $x_{i}$ and $x_{j}$ are the vectors of the concatenated rest profiles for subjects i and j, $d(x_{i},x_{j})\;$is the distance between these two vectors, and $n_{m}$ is the number of subjects in cluster $C_{m}.\;$We computed the Euclidean distance of these pairs of vectors, i.e. the root of the squared differences between their components. The number of clusters for each shift condition was guided by the ‘elbow’ method,^43^ which plots a measure of dissimilarity, here $W_{k}$, against the number of clusters k. As k increases, $W_{k}$ decreases monotonically, but usually ‘flattens’ from some k onwards, which is used as the number of clusters, and the corresponding division is taken as the estimated cluster membership.

#### Multivariate regression analyses

Multivariate regression analyses were used to investigate the effect of shift type and other characteristic covariates from questionnaire, i.e. age, chronotype, and years of night work, and possible pairwise interactions, on the four circadian parameters of interest, namely p1-1 as a measure of the uninterruptedness and thus quality of rest, RI as a measure of the regularity and quality of rest, the estimated rest amount as an indicator of the quantity of rest, and the estimated rest centre time as a measure of the phase of the biological clock. Since p1-1 is a probability, the log odds ratio $\log(\frac{p1-1}{1-p1-1})$ (LOP1-1) was used in the regression analyses. It maps the interval [0,1] onto the real line with increasing values corresponding to an increasing p1-1 and quality of rest. As RI also varied between 0 and 1, the log odds ratios, $\log(\frac{RI}{1-RI})$ (LORI) was used instead of RI. The pairwise multiplicative interaction terms capture the simultaneous effect of two covariates. For example, an interaction between age and shift type suggests that the effect of age is different between DS and NS.

A bivariate copula additive model^44^ was assumed for the response variable which is bivariate in that each subject displays a response to work and free days in each of the circadian parameters. A copula is a function used to link multiple univariate marginal distributions of random variables into a multivariate distribution (see^45^ for a review while a list of examples of copula functions can be found in Table S5). We assumed that the mean of the marginal distribution of the response variable, i.e. a circadian parameter, on work and free days is linked via a link function to a predictor, which is a linear combination of covariates and interaction terms. The means of the two marginal response distributions, *μ_work_* and $\mu_{free}$, were hence expressed as

$$\left\{ \begin{array}{c} g_{\mu_{work}}\left(\mu_{work}\right)=\eta_{work}=\beta_{0}^{1}+\beta_{1}^{1}*ShT+\beta_{2}^{1}*Age+\beta_{3}^{1}*CS+\beta_{4}^{1}*YNW+\;\;\beta_{5}^{1}*ShT*Age+\;\beta_{6}^{1}*ShT*CS+\;\beta_{7}^{1}*ShT*YNW+\;\beta_{8}^{1}*Age*CS+\beta_{9}^{1}*Age*YNW+\beta_{10}^{1}*CS*YNW\\ g_{\mu_{free}}\left(\mu_{free}\right)=\eta_{free}=\beta_{0}^{2}+\beta_{1}^{2}*ShT+\beta_{2}^{2}*Age+\beta_{3}^{2}*CS+\beta_{4}^{2}*YNW+\;\;\beta_{5}^{2}*ShT*Age+\;\beta_{6}^{2}*ShT*CS+\;\beta_{7}^{2}*ShT*YNW+\;\beta_{8}^{2}*Age*CS+\beta_{9}^{2}*Age*YNW+\beta_{10}^{2}*CS*YNW \end{array}\right.$$

where $g_{\mu_{work}}\left(\cdot \right)$ and $g_{\mu_{free}}\left(\cdot \right)$ are the link functions for $\mu_{work\;}$ and $\mu_{free}$; $\eta_{work}$ and $\eta_{free}$ are the predictors; ShT is the shift type (0=day shift, 1=night shift); Age (values between 22 and 62) is the age in years; CS (values between 24 and 74) is the chronotype score with larger values representing more morningness; YNW (values between 0 and 35) is the years of past night work; ShT*Age, ShT*CS, ShT*YNW, Age*CS, Age*YNW, and CS*YNW are interaction terms; and the β‘s are regression coefficients including an intercept. Note that in contrast to performing two separate regressions, the formulation of the two response variables via a bivariate distribution allows to model the dependence between the responses for work and free days, which are measured on the same subject. This is addressed by the copula additive model that binds the marginal distributions with a copula function whose parameter θ measures dependence between the marginals.^45^ The copula additive model is very flexible and can allow for various distributions in addition to the Gaussian model. Our choices of distributions and details of the model selection, which was based on the corrected Akaike Information criterion AICc^13^ and standard methods for residual analysis, can be found in SM-6.

#### Spectral analysis

The Spectrum-Resampling (SR) algorithm^46^ was applied to the hourly means of Chesttemp over the monitoring period to estimate spectral densities and 90% confidence intervals for the period lengths identified as spectral peaks. The dominant period corresponds to the largest peak. Participants were grouped by their dominant periods in order to investigate the effect of shift work on circadian temperature rhythm. The gravity centre of the area under the individual spectral density curve was calculated as a summary statistic for the spectrum. As individuals generally have spectral peaks at 12h and/or 24h, a value of the gravity centre between 12h and 24h indicates that an individual displays both these periodicities. Furthermore, motivated by,^47^ spectral analysis was also performed on 5-min Chesttemp and square root of 5-min PA with the aim of detecting possible ‘ultradian’ periodicities (<12 hours) during rest bouts. An inverse transformation was applied to PA data amplifying low activity, or Locomotor Inactivity During Sleep (LIDS),^47^ making it easier to detect cyclicity of inactivity during time spans of small movement. We defined a rest bout as the time span (considering those between 2h and 12h) of an individual's rest state estimated by the 2-HHMM, and investigated the presence of ultradian fluctuations in Chesttemp and LIDS PA and any shift work effects.

#### Procedure of analysis and statistical test

**Figure 1:**
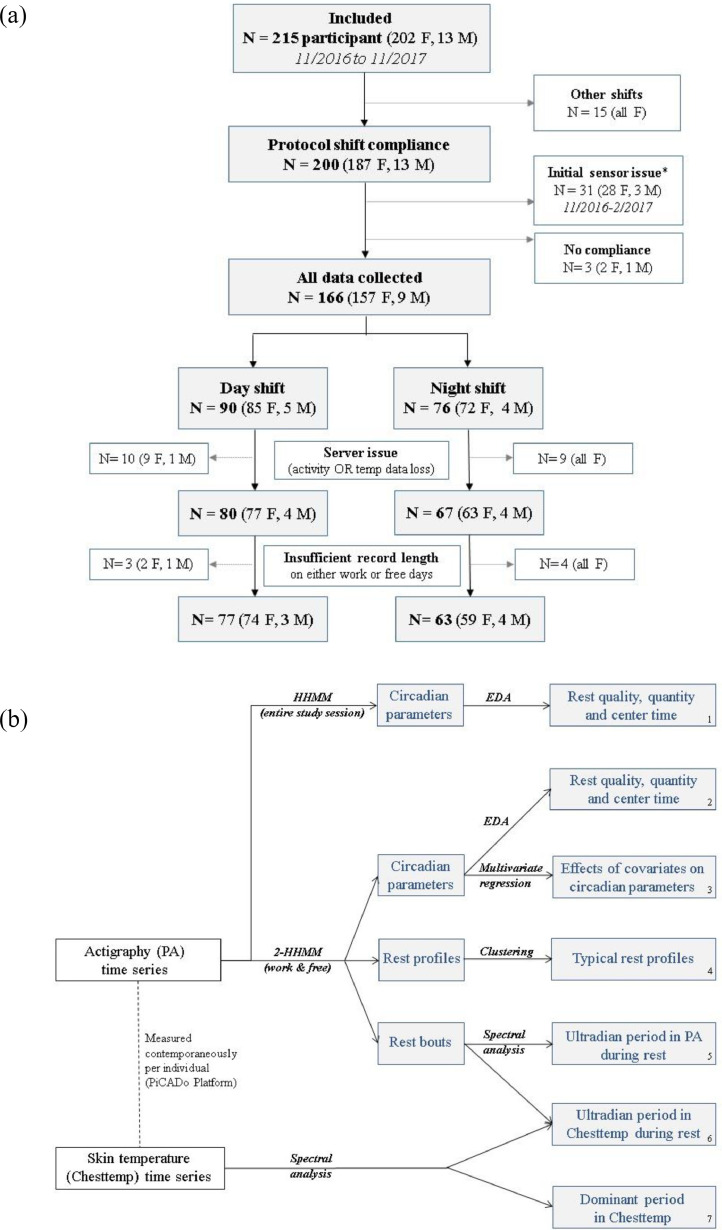
**a: Consort diagram**. Consort diagram showing the enrolment of subjects in the study and selection of subjects whose records that were adequate on both work and free days for analysis. Subjects are stratified according to shift type (day shift or night shift). No participant alternated day and night shift. *Heat-related disconnection of electronic cards within the GPRS gateway. **b: Analysis Chart**. The template for the statistical analysis of multidimensional data sets including physical activity and skin temperature. ‘EDA’ denotes the exploratory data analysis.

Our analysis proceeded in the following order: The PA and Chesttemp time series over the entire study session were first analysed. The HHMM approach captured the specificities in the observed temporal rest-activity patterns of each individual DS or NS during the whole study span, and summarised them with the four selected circadian parameters (block 1 in Figure 1b). Spectral analysis was applied to estimate the main rhythmic properties of Chesttemp measured simultaneously with the same sensor (block 7 in Figure 1b). The entire study session was then divided into work and free days for every individual according to their diary. The 2-HHMM was then fitted to the PA data and the circadian parameters on work and free days were calculated and compared (block 2 in Figure 1b). A subsequent unsupervised cluster analysis of the individual daily rest profile from the fitted 2-HHMM identified distinct subgroups with similar rest patterns within the DS and NS groups (block 4 in Figure 1b). These clusters were then compared with respect to other circadian and ultradian parameters in PA and Chesttemp. A multivariate regression approach with bivariate response – to accommodate work and free days – investigated the effect of the shift work schedule and other covariates, such as age, chronotype score, and the number of years of prior night work, on the circadian parameters (block 3 in Figure 1b). Finally, spectral analysis was performed on subsections of the PA and Chesttemp corresponding to rest bouts (as previously identified by the 2-HHMM) to estimate ultradian subperiodicities. We analysed their correlation between PA and Chesttemp and compared period lengths between DS and NS (blocks 5 and 6 in Figure 1b).

Many variables, including the estimated circadian parameters, mean activity levels of the three states, and ultradian subperiods, did not appear to follow a Gaussian distribution, so we turned to the use of nonparametric techniques such as the Mann-Whitney U test to determine whether DS and NS nurses had different value distributions. The Wilcoxon signed-rank test was used to compare the values of circadian parameters on work and free days. The correlation of single circadian parameters between work and free days, and the correlation between ultradian subperiods of PA and Chesttemp were investigated via the Spearman correlation test. Comparisons between clusters were conducted by the Kruskal-Wallis test. The significance of a covariate effect in the regression analysis was assessed by the p-value of the approximate two-sided z-test.^44^ Statistical analysis was performed in R.

### Role of funders

Funding sources had no role in the conduct or reporting of the research.

## Results

### Participants’ characteristics and study conduct

The Circadiem study recruited 215 health professionals (Figure 1a), including 140 working on a standardized day or night shift schedule, who provided adequate longitudinal data on work and free days (supplementary Table S1). The 140 participants aged between 22 and 62, and 95% of them were female. The range of BMI was from 18 to 40. NS were significantly older (44·1 vs 37·5 years old), with more post-menopausal participants (44% vs 18%), as compared to DS. Questionnaires and diaries showed that NS had significantly shorter nocturnal sleep duration on workdays than DS (5·0 h vs 6·8 h from questionnaires; 5·2 h vs 6·7 h from diaries), but both groups had similar sleep duration on free days. NS had a mean sleep debt^34^ that was twice as high (2·2 h vs 1·0), and significantly later sleep midpoints on both work and free days, lower morningness-eveningness scores (47·3 vs 53·3), and fewer morning chronotypes compared to DS. The median percentage of missing values in both, physical activity (PA) and chest temperature (Chesttemp), was only 2·76% (IQR 1·58% to 5·24%).

### Circadian parameters and chest temperature over the entire study session

**Figure 2:**
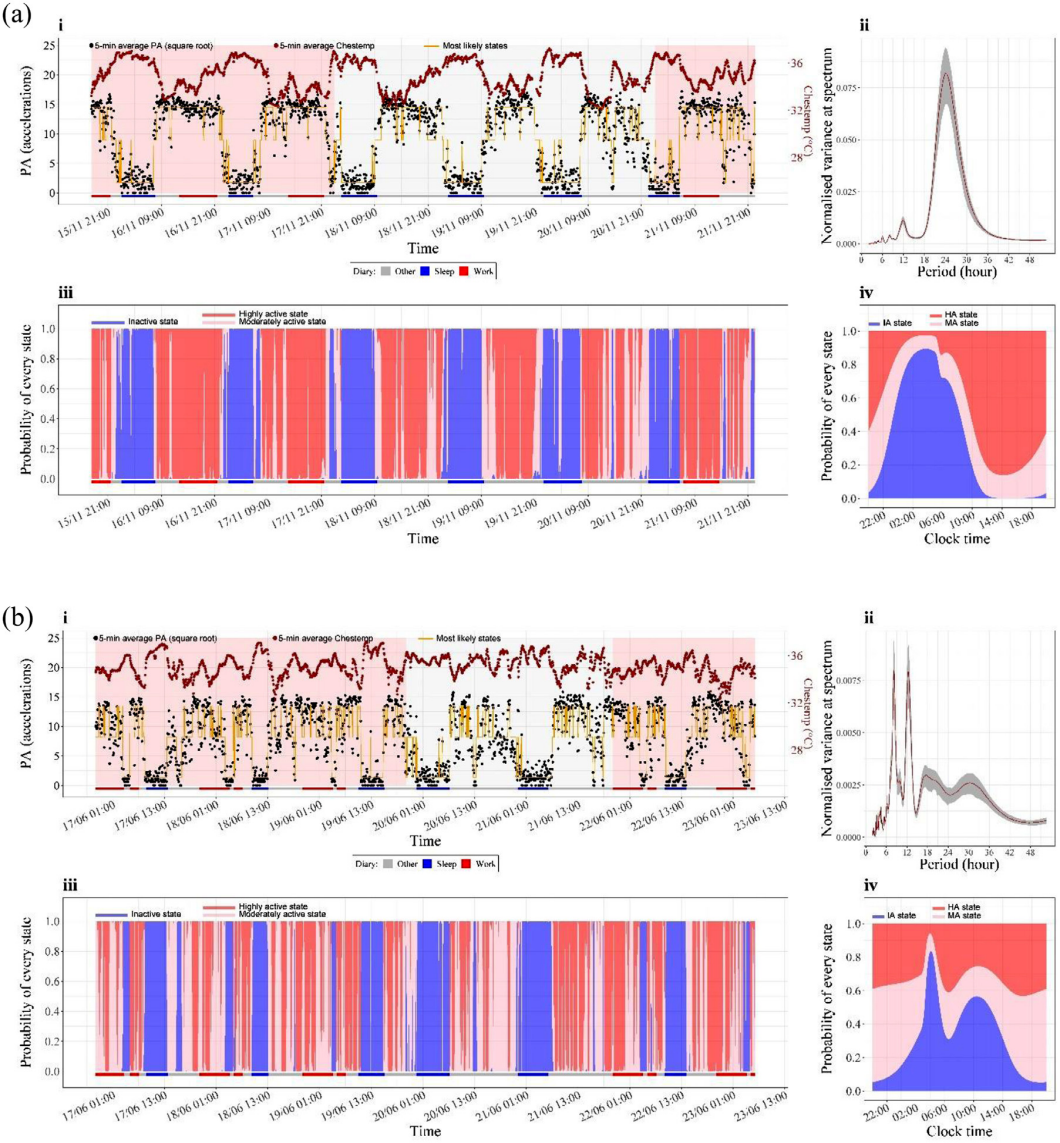
**a: State estimation and spectral analysis for subject 1219 (39 y.o. female, DS)**. **b: State estimation and spectral analysis for subject 1363 (45 y.o. female, NS)**. (**i**) Time series of PA (black dots) and Chesttemp (brown dots) with yellow line indicating the most likely state using local decoding. The red and blue horizontal bars denote the work and sleep periods recorded in the diary. This plot shows that the rest state dominated the sleep period recorded in diary and that there were a lot of transitions between the MA and HA state during the remaining monitoring period. (**ii**) Spectral density estimates of Chesttemp (brown line) with respective 90% confidence intervals (grey area). (**iii**) State probability plot during the whole study period, i.e. cumulative plot of $\text{Pr}(S_{t}=j|\;O^{\left(T\right)})\;$for j = 1 (IA, blue), 2 (MA, pink), 3 (HA, red). It allows a quick assessment of how probable the most likely state is and what other states have noticeable probability and provides visual information on how well a subject has rested.^31^ In particular, if they have solid blue areas, i.e. rarely move into the active states during rest, then it could be summarized that this subject has obtained a good rest, as the subject 1219 has done. (**iv**) Circadian state probability plot from HHMM obtained as the averaged probability of every state over the whole study period. It shows the periodic time profile of the three state probabilities plotted in cumulative manner analogous to (**iii**) with same colour coding as in (**iii**). The blue area represents the probability of rest and gives the daily profile of rest. Subject 1219 had one rest every day shown by **a(iv)** while subject 1363 was likely to rest twice in one day illustrated by two peaks in the rest profile in **b(iv)**.

**Figure 3:**
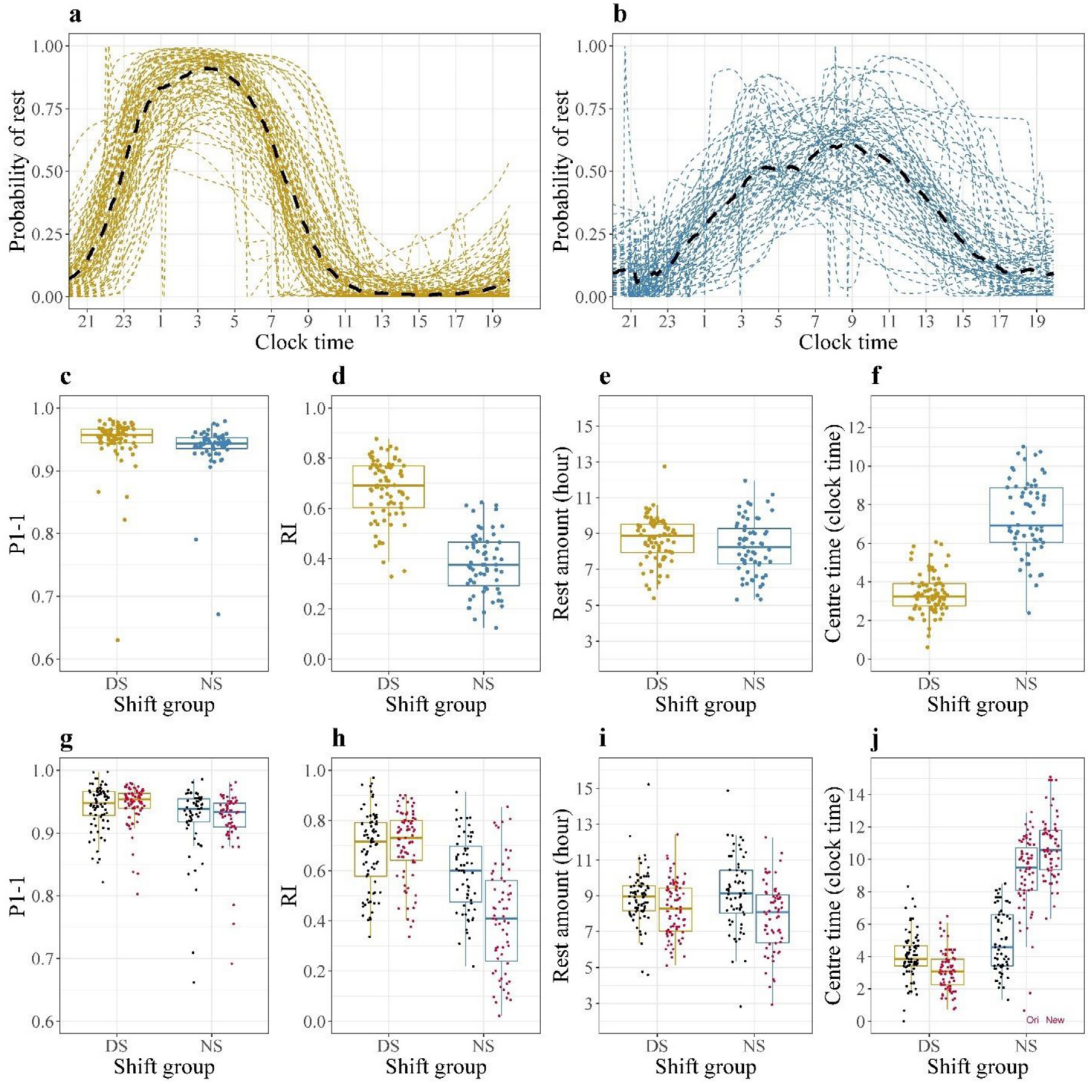
**Daily profile of rest for two shift groups and boxplot of individual circadian parameters**. (**a**-**b**) Plots of probability of rest for all 77 DS (yellow dashed lines) with group median (black dashed line) in left panel and for all 63 NS (blue dashed lines) with group median (black dashed line) in right panel. The individual probability of rest line is the same as the boundary of blue part in Figure 2**a(iv)** and **2b(iv)**. The group median is obtained by taking the median of probabilities of rest for all members at each time point. (**c**-**f**) Plots of the four circadian parameters during the whole study period stratified by shift type. Boxes with yellow dots are for DS, boxes with blue dots are for NS. The median of individual p1-1 for DS is 0·96 [IQR, 0·94-0·97] while for NS it is 0·94 [0·94-0·95]. The median of individual RI for DS is 0·69 [0·60-0·77] while for NS it is 0·38 [0·29-0·47]. The median of individual rest amount for DS is 8·9 [7·9-9·5] while for NS it is 8·2 [7·3-9·3]. The median of individual centre time of rest for DS is 3·3 [2·8-3·9] while for NS it is 6·9 [6·0-8·9]. (**g**-**j**) Plots of the four circadian parameters on workdays and free days separately stratified by shift type. Yellow boxes are for DS, blue boxes are for NS. Within each shift type, boxes with black dots (left) represent free days, boxes with red dots represent workdays (right). Take (**g**) as an example. Comparing the two boxes with black (red) dots is to compare the p1-1 on free (work) days between DS and NS. Comparing the left (right) two boxes is to compare the p1-1 between work and free days for DS (NS). In (**j**), the original value of centre time denoted by ‘Ori’ is the gravity centre of the area under the probability of rest curve, while the new value (‘New’) is the timing of the maximum probability of rest between 07:00 and 20:00. Numerical representation of boxplots can be found in Table S2A-B.

The majority of DS had clear 24-hour rhythmic patterns in PA and Chesttemp, on work and free days (Figure 2a). Spectral analysis revealed a clear peak in Chesttemp at the frequency corresponding to a 24-h period. Some DS showed prominent 12-h harmonics in Chesttemp (Figure S3a). In contrast, the majority of NS had non-circadian or abnormal rhythmic patterns in PA and Chesttemp (Figure 2b, S3b). Daily rest profiles were homogeneous within the DS group but subject to variation within the NS group (Figure 3a-b). The median value of the maximum of the rest profile, corresponding to the maximum probability of being in rest state, was 0·91 at 03:20 at night for DS as compared to 0·61 at 08:55 for NS (Figure 3a).

NS had lower mean values of the high (p=0·03) and moderate activity states (p=0·0015), lower p1-1 and Rhythm Index (RI) (both p<0·0001), a delayed centre time of rest (p<0·0001) and a shorter rest amount (p=0·061) (Figure 3c-f, tests based on Mann-Whitney U-test) than DS. Thus, night shiftwork affected not only the quality of rest, estimated by p1-1, and its rhythmicity, estimated by RI, but also the amount and timing of rest.

**Figure 4:**
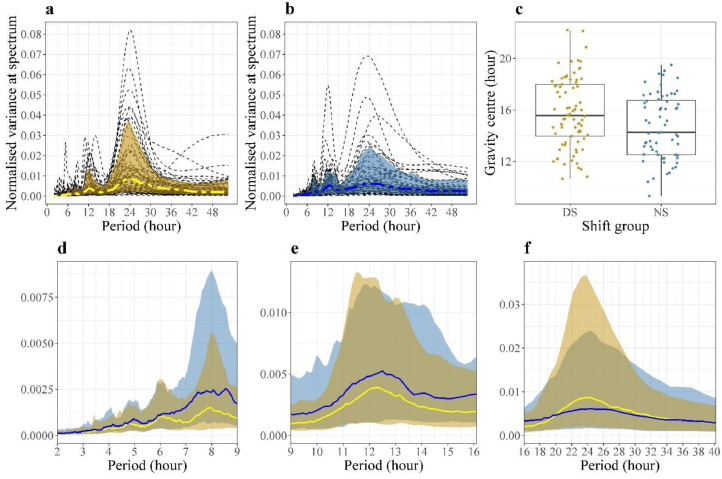
**Spectral density estimation of Chesttemp for two shift groups**. (**a**-**b**) Plots of spectral density estimates (black dashed line) for every subject with group median (blue/yellow dashed line) and 10%-90% quantile (blue/yellow area). The individual spectral density estimate line is the same as Figure 2**a(ii)** and **2b(ii)**. The group median is obtained by taking the median of the densities for all DS/NS. The lower and higher boundary of the light blue/yellow ‘envelope’ are equal to the 10^th^ and 90^th^ percentiles across the individual densities, respectively. These ‘envelopes’ provide an idea of where most individual densities are. Besides the dominant 24h rhythm, a dominant circa-12h rhythm, with a period ranging from 10h to 14h, was identified in 12 DS (16%) and 20 NS (30%). There were 2 DS (3%) and 6 NS (10%), who displayed a dominant period around 8h (7h-9h), and a further 9 DS (12%) and 8 NS (13%) with other dominant or undetectable periods. (**C**) Boxplots of the spectral gravity centres across all individuals. The median gravity centre for DS is 15·6h [IQR, 14·0 to 18·0] and for NS is 14·3h [12·5 to 16·8]. (**d**-**f**) Magnifications of the plots (**a**-**b**) showing group median estimates (yellow for DS, blue for NS) and 10%-90% quantile (yellow for DS, blue for NS) in three intervals (2-9h, 9-16h, 16-40h). A larger proportion of DS had a 24h dominant period, resulting in a sharp peak of the group median line and the group envelope in (**a**) and (**f**). The flatter and wider peak at 24h for NS group illustrates more variability in the dominant periods around 24h in (**b**) and (**f**). The magnified plots in (**d**) highlight the fact that NS had more pronounced spectral peaks at shorter periods, especially around 8h.

A circadian rhythm was found for Chesttemp in 57 DS (70%) and 32 NS (48%) (**χ^2^**, p=0·027) (Figure 4). The NS tended to display shorter periods than DS as summarised by the gravity centre of the spectral density (14·7 vs 15·8; **χ^2^**, p=0·025; Figure 4c). In particular, 49% and 62% of the individual spectral densities for DS and NS, respectively, had a lower peak around 8h (Figure 4d).

### Circadian parameters on work versus free days

Based on the 2-HHMM, the maximum of the daily rest probability of the individual NS ranged between 06:20 and 15:05 (Figure 3j). NS had lower p1-1, lower RI and a later centre time of rest than DS both on workdays (Mann-Whitney U test, p<0·0001 for all) and free days (p<0·02 for all) (Figure 3g-j; Table S2b-c). Both, DS and NS, rested for a longer time on free days compared to workdays (Wilcoxon signed-rank test, p=0·0010, p<0·0001), yet without any significant difference in the amount of rest between workdays (Mann-Whitney U test, p=0·14) and free days (p=0·47). The centre time of rest remained delayed in NS compared to DS on free days (Mann-Whitney U test, p=0·0013). The RI of NS increased on free days compared to workdays (Wilcoxon signed-rank test, p<0·0001) but still displayed significantly worse values compared to DS on both work and free days (Mann-Whitney U test, p<0·0001, p=0·0005). For both groups, positive values of Spearman correlations supported the consistency of circadian rest-activity parameter values between work and free days (SM-3) within individuals.

### Clustering of individual daily rest profiles

**Figure 5:**
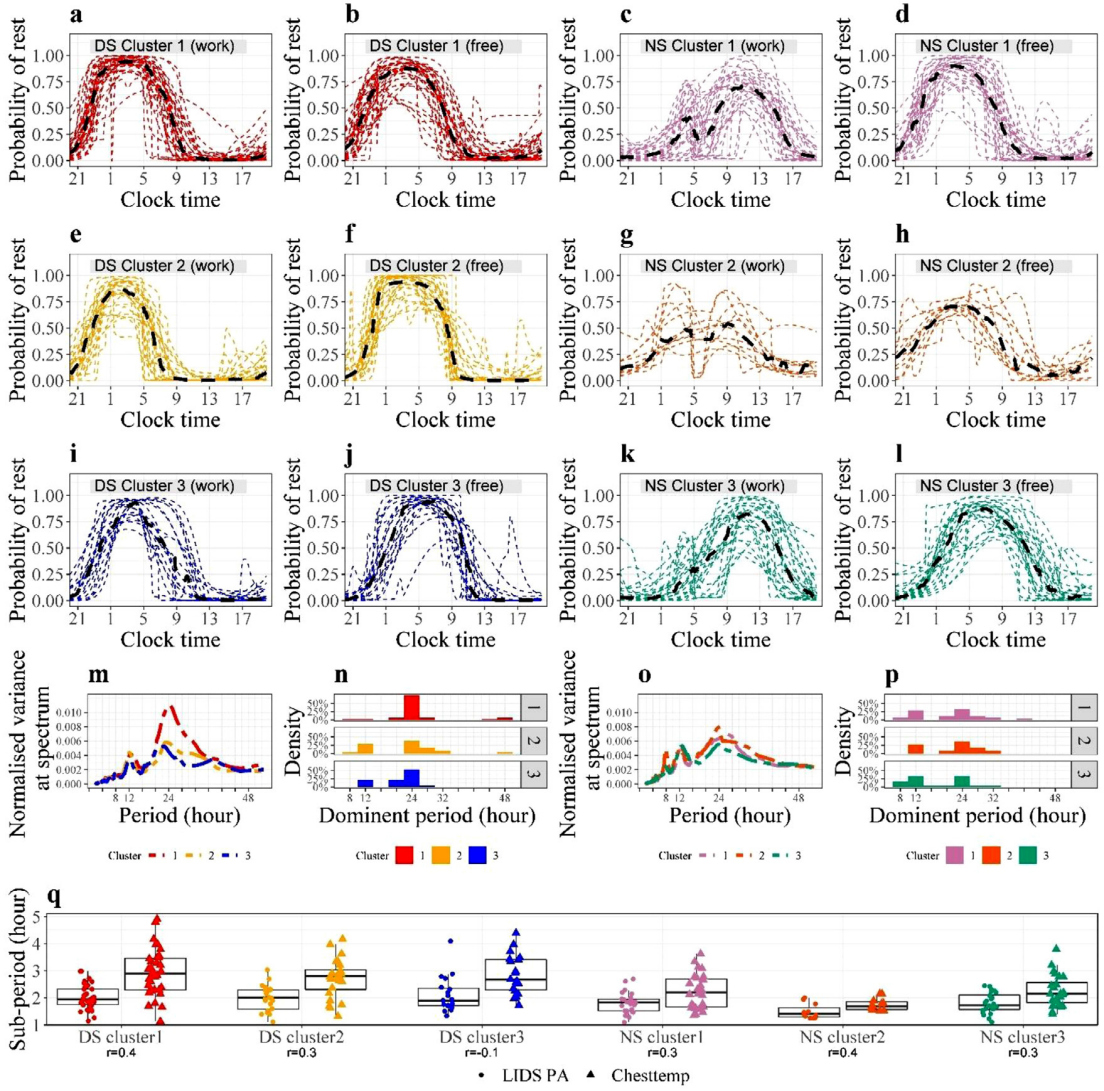
**Three DS clusters and three NS clusters**. (**a**) (**b**) (**e**) (**f**) (**i**) (**j**) Plots of probability of rest for all DS (cluster 1: red dashed lines; cluster 2: yellow dashed lines; cluster 3: blue dashed lines) with cluster median (black dashed lines), similar to Figure 3**a**. The cluster median is obtained by taking the median of probabilities of rest for all members at every time point. There are 34 (44%), 24 (31%), and 19 (25%) subjects in cluster 1, 2 and 3 respectively. (**c**) (**d**) (**g**) (**h**) (**k**) (**l**) Plots of probability of rest for all NS (cluster 1: purple dashed lines; cluster 2: orange dashed lines; cluster 3: green dashed lines) with cluster median (black dashed lines) similar to Figure 3**b**. The cluster median is obtained by taking the median of probabilities of rest for all members at every time point. There are 28 (44%), 11 (17%), and 24 (38%) subjects in cluster 1, 2 and 3 respectively. (**m**) (**o**) Plot of cluster median obtained by taking the median of the densities for all DS/NS subjects in one cluster. (**n**) (**p**) Histogram of dominant period in Chesttemp for three DS/NS clusters. (**q**) Boxplot of individual sub-periods for every cluster. The individual sub-period is the median of sub-periods over several rest bouts. The labels underneath the x_axis denote the Spearman correlation coefficient between sub-periods of LIDS PA and Chesttemp.

Three clusters, each containing between 11 and 34 participants, were identified for each shift work group (Figure S4). All three clusters belonging to DS had a unimodal circadian rest profile indicating monophasic sleep on both work and free days (Figure 5a-b, e-f, i-j)**.** The four circadian parameters differed significantly between clusters, both on work and free days, except for the rest amount during free days (Table S3a). DS in cluster 1 had higher average RI on workdays, and lower p1-1 and RI on free days, compared to both other clusters. This finding suggested their stronger circadian synchronization on dayshift work compared to free days. DS in cluster 2 had the lowest mean p1-1 and RI during workdays, and the highest mean RI when they were off work, indicating a poor fit between their circadian clocks and the work schedule. DS in cluster 3 had the highest p1-1 and latest centre time of rest, both on work and free days, suggesting a more immediate adjustment of their circadian clocks to changes in daily life schedules.

The circadian rest profiles of NS were markedly more heterogeneous (Figure 5c-d, g-h, k-l). On workdays, the 24-h rest pattern was bimodal for the majority of the NS in clusters 1 and 2 (64% and 73% of the NS, respectively), where the first rest bout occurred around 04:25 and 03:05, respectively, i.e. during night work, and a second one was taken around 11:25 and 08:55, respectively, i.e. after night work (Figure 5c, g). The rest profile was unimodal for 67% of the NS in cluster 3 indicating that they had a single rest span during workdays (Figure 5k). On free days, all NS followed a unimodal rest profile with the peak times of rest occurring earlier for clusters 1 and 2 (03:15 and 03:50, respectively; Figure 5d, h) as compared to cluster 3 (06:45; Figure 5l). The RI was worst both on workdays and on free days for the NS in cluster 2 (Kruskal-Wallis test, p=0·0033), whose centre time of rest occurred the earliest on workdays (p<0·0001) (Table S3b). Also Chestemp patterns differed between DS and NS clusters. For DS, the Chesttemp spectra peaked solely at 24h for cluster 1, but at both 24h and 12h for clusters 2 and 3 (Figure 5m-n). For NS, the dominant Chesttemp periods were similar for all three clusters (Figure 5p), although subjects in NS cluster 2 also had a marked 8h-harmonic period in Chesttemp (Figure 5o).

### Predictors of circadian parameters

Multivariate regression analysis revealed that shiftwork type and age were significant predictors of p1-1 on workdays, whilst shift work type was the single predictor of p1-1 on free days. Night shift work adversely affected the rest quality estimated by p1-1 on both work and free days (z-test, p*<*0·0001, p=0·0098) (Table 1). Age had an adverse effect on p1-1 on workdays (z-test, p=0·0089), i.e. older subjects were likely to have lower p1-1, thus a poorer rest quality on workdays (Figure S5a). Further analysis highlighted the key role of the number of years of night work as a predictor of RI, rest amount and centre rest time. The number of years of night work had an adverse effect on RI for both DS and NS on both work (z-test, p=0·0074) and free days (z-test, p=0·0005) (Figure S6c, d). Age, chronotype, and shift work type were additional predictors of RI on workdays but not on free days. Age had a positive effect on RI for DS on workdays (z-test, p=0·039) (Table 1), i.e. older DS had a higher RI on workdays than younger DS. The opposite was found for NS, as their RI on workdays deteriorated with increasing age (Figure S6a). The chronotype score was positively correlated with RI for DS, suggesting that DS with higher morningness scores had higher RI on workdays. In contrast, NS with higher morningness scores had lower RI on workdays.

**Table 1:** Parametric coefficients in the regression models.

Parametric coefficients	Estimate	Std.Error	z-value	Pr(>|z|)	
Log odds p1-1 on workdays					
$\beta_{0}^{1}\;$(intercept)	3·335	0·128	25·987	<0·0001	***
$\beta_{1}^{1}$ (night shift)	-0·350	0·073	-4·783	<0·0001	***
$\beta_{2}^{1}$ (age)	-0·009	0·003	-2·614	0·0089	**
Log odds p1-1 on free days					
$\beta_{0}^{2}$ (intercept)	2·951	0·062	47·443	<0·0001	***
$\beta_{1}^{2}$ (night shift)	-0·242	0·094	-2·581	0·0098	**
Log odds RI on workdays					
$\beta_{0}^{3}$ (intercept)	-3·158	1·471	-2·148	0·0318	*
$\beta_{1}^{3}$ (night shift)	2·863	0·936	3·058	0·0022	**
$\beta_{2}^{3}$ (age)	0·075	0·036	2·068	0·0386	*
$\beta_{3}^{3}$ (chronotype score)	0·090	0·028	3·183	0·0015	**
$\beta_{4}^{3}$ (years of night work)	-0·202	0·075	-2·679	0·0074	**
$\beta_{5}^{3}$ (night shift: age)	-0·065	0·014	-4·462	<0·0001	***
$\beta_{6}^{3}$ (night shift: chronotype score)	-0·028	0·014	-2·000	0·0455	*
$\beta_{7}^{3}$ (age: chronotype score)	-0·002	0·001	-2·453	0·0142	*
$\beta_{8}^{3}$ (age: years of night work)	0·004	0·001	2·847	0·0044	**
Log odds RI on free days					
$\beta_{0}^{4}$ (intercept)	0·815	0·076	10·767	<0·0001	***
$\beta_{1}^{4}$ (years of night work)	-0·034	0·010	-3·476	0·0005	***
Rest amount on workdays					
$\beta_{0}^{5}$ (intercept)	2·389	0·066	36·425	<0·0001	***
$\beta_{1}^{5}$ (night shift)	0·158	0·121	1·305	0·1920	
$\beta_{2}^{5}$ (age)	-0·006	0·002	-3·320	0·0009	***
$\beta_{3}^{5}$ (years of night work)	0·011	0·003	3·609	0·0003	***
$\beta_{4}^{5}$ (night shift: age)	-0·006	0·003	-1·923	0·0545	.
Rest amount on free days					
$\beta_{0}^{6}$ (intercept)	2·521	0·074	33·916	<0·0001	***
$\beta_{1}^{6}$ (age)	-0·005	0·001	-3·743	0·0002	***
$\beta_{2}^{6}$ (chronotype score)	-0·002	0·001	-1·724	0·0847	.
$\beta_{3}^{6}$ (years of night work)	0·007	0·002	3·782	0·0002	***
Rest centre time on workdays					
$\beta_{0}^{7}$ (intercept)	2·429	0·215	11·297	<0·0001	***
$\beta_{1}^{7}$ (night shift)	-0·118	0·303	0.390	0·6962	
$\beta_{2}^{7}$ (age)	-0·006	0·003	-2.504	0·0123	*
$\beta_{3}^{7}$ (chronotype score)	-0·018	0·004	-4·748	<0·0001	***
$\beta_{4}^{7}$ (night shift: age)	0·008	0·004	1·952	0·0510	.
$\beta_{5}^{7}$ (night shift: chronotype score)	0·013	0·005	2·673	0·0075	**
Rest centre time on free days					
$\beta_{0}^{8}$ (intercept)	7·560	1·119	6·758	<0·0001	***
$\beta_{1}^{8}$ (night shift)	4·982	1·343	3·708	0·0002	***
$\beta_{2}^{8}$ (age)	-0·025	0·013	-1·536	0·1245	
$\beta_{3}^{8}$ (chronotype score)	-0·051	0·019	-2·663	0·0077	**
$\beta_{4}^{8}$ (years of night work)	-0·367	0·121	-3·042	0·0023	**
$\beta_{5}^{8}$ (night shift: chronotype score)	-0·080	0·025	-3·151	0·0016	**
$\beta_{6}^{8}$ (age: years of night work)	0·007	0·002	3·054	0·0022	**

Significance codes: 0 ‘***’ 0·001 ‘**’ 0·01 ‘*’ 0·05 ‘.’ 0·1 ‘ ’ 1.

The number of years of night work also predicted rest amount (RA) on workdays, jointly with shift-work type and age, and their interaction, and on free days, jointly with age and chronotype score. For both shift-work types, older subjects had less rest on either work (z-test, p=0·0009) or free days (p=0·0002) (Table 1; Figure S7a, d). Prior exposure to night shift work also affected rest amount where subjects with more years of night work took longer rests on workdays (z-test, p=0·0003) as well as free days (p=0·0002) (Figure S7c, f).

Finally, the number of years of night work also had a significant impact on the rest centre time, jointly with age, chronotype, and shift work type, along with the interaction of the latter, on free days (Table 1; Figure S8). The negative correlation between rest centre time and chronotype score was affected by the night shift work (z-test, p=0·0075 and p=0·0016, respectively).

### Ultradian oscillations in PA and Chesttemp during rest

A total of 2,338 rest bouts, of which 1,111 lasted 2 to 12 h, were selected, with 555 for DS and 556 for NS. The distribution of the ultradian sub-periods estimated for LIDS PA and Chesttemp were both right-skewed each with a mode around 1·5h (Figure S9a-b) and central probability mass around 1h to 2h. A larger proportion of sub-periods for PA were around 1·5 h (Figure S9a), while the distribution of Chesttemp sub-periods had a longer right tail (Figure S9b). The ultradian period lengths of both LIDS PA and Chesttemp were significantly shorter for NS than for DS (LIDS PA: 1·8h vs 2·2h; Chesttemp: 2·3h vs 2·9h; p*<*0·0001 for Mann-Whitney U test) (Figure S9c-d). The ultradian period lengths of LIDS PA and Chesttemp were strongly correlated within subjects, both in the whole study population and for each group (Spearman correlation: all, r=0·5281; DS, r=0·4065; NS, r=0·5873; p*<*0·0001 for each comparison to zero correlation) (see an illustrative example in Figure S10). Ultradian period lengths were similar between the three DS clusters, but differed significantly between the three NS clusters for both LIDS PA (Kruskal-Wallis test, p=0·0009) and Chesttemp (p=0·0013) with cluster 2 having the shortest ultradian sub-periods (Figure 5q).

## Discussion

Longitudinal telemonitored data of high quality were gathered from 140 health professionals working as DS (77 individuals) or NS (63 individuals). The distribution of the circadian parameters in rest-activity and chest surface temperature of the DS in Circadiem was consistent with earlier results from telemonitoring “day-active” people using the same system,^30^^,^^48^^,^^49^ which further highlights the significance of the alterations found in the NS. Indeed, NS experienced significantly worse estimated rest quality (p1-1) and circadian activity rhythm (RI) and a delayed centre time of rest, although they achieved similar rest duration by taking more frequent and longer naps. This finding is consistent with prior studies^50^ using diaries or wrist actigraphy. Both NS and DS extended rest duration on free days to compensate for the loss of sleep on workdays (indicated by increased rest amount on free days). However, NS suffered from lower rest quality estimated by p1-1 on work and free days. Although some re-adjustment could be discerned, the delay in rest centre persisted significantly on free days. These findings indicate that the actual length of free time following night shift was insufficient to ensure recovery of circadian rhythms in the NS participants.^51^^,^^52^

Ultradian oscillations, with about-2h periodicities, were identified using wrist accelerometry during sleep, and were linked to sleep depth and alternations in Rapid-Eye-Movements sleep stages in . Here, we confirmed such ultradian periodicities during sleep, using a thoracic rather than a wrist accelerometer, and discovered strongly correlated ultradian period lengths in both chest accelerometry and surface temperature during sleep. We also found that NS had 22% shorter periodicities than DS for both variables, thus supporting a non-invasive and quantitative approach to investigate and correct sleep alterations in NS, and their links to circadian disturbances.

Clustering analysis categorized the between-subject differences in responses to repeat day or night shifts. The daily rest profile of NS on workdays was either bimodal, with two periods of rest, or unimodal. A cluster was revealed containing 11 NS (17%) with a bimodal rest pattern and lowest values for p1-1 and RI on free and workdays, thus displaying the poorest adaptation of their circadian timing system to the night shift work schedule. We hypothesized that the NS in this cluster were suffering from severe social jet lag on night shift work, since they were predominantly of ‘morning oriented’ chronotype. Compared to the NS nurses in the other clusters, they had shorter ultradian periodicities in activity and temperature during sleep.

Multivariate analysis confirmed that night shift work significantly predicted poor estimated sleep quality (p1-1). Most importantly, this analysis revealed that more years of night work predicted lower RI and shorter rest duration on both work and free days, and centre time of rest occurring later on workdays and earlier on free days. Age and chronotype score were also influential on RI, rest amount, and centre rest time. The prediction that years of night work are affecting circadian rest-activity and temperature parameters were intriguing considering their reported relationship with increased risk of breast cancer and cardiovascular diseases.^5^^,^^7^^,^^53^ Thus, the biomarkers identified here might constitute early warning signals of poor health and subsequent risk of life-threatening disease, which could guide medical interventions and follow-up.

Limitations of the study arise from the fact that the study span lasted a single week with no planned confirmatory session, and the lack of planned medical follow-up. An initial technical issue occurred removing a random set of participants unrelated to their characteristics, hence reducing the sample size without altering the study design. While the number of male participants was small, reflecting the sex ratio in the nursing profession, the proportion of males was about the same in the day shift and the night shift groups in line with a balanced design. However, while we checked that outliers were not systematically associated with male participants, a sex effect could not be tested and this places a limit on the interpretation of our results. As per inclusion criteria, our study could not involve participants with intolerance to night work before recruitment, who would have quickly dropped off from night work. Future studies in this area should also determine digital circadian and sleep health and their relations with prior cumulated night work exposure (i) in male night or day shift workers; and (ii) from the start of employment, for those professionals, for whom night shift work represents a frequent option.

Our new telemonitoring and artificial intelligence methodology and the findings we made here highlight a potential path toward individual occupational risk assessment. The Circadiem study results support the imprinting of circadian rhythm and sleep disruptions in association with prolonged night work, a main determinant of cancer and cardiovascular risk. In the future, this methodology could become integral to routine occupational medicine to detect increased risk based on circadian and ultradian disruption and support interventions to the work schedule of an individual at risk. Large-scale epidemiological studies would further assess the relevance of individual circadian disruption exposure to risks of cancers and cardiovascular diseases. Toward this goal, our technology has recently been automatized for facilitating real-time decision making in a clinical trial involving the telemonitoring of circadian and sleep cycles in pancreatic cancer patients (ClinicalTrials.gov Identifier: NCT04263948). This trial is also paving the way for ethical and GPDR considerations when applying telemonitoring in field studies. These include participants’ understanding of the technology system and expected outputs, as well as their engagement and training as a user. The deidentification of time series records and automated analyses is needed to protect privacy, with encoding only known to a physician-investigator. Teletransmitted data need to be stored and automatically analysed within a national regulatory-approved health data hub. Participant-approved scientists, physicians, nurses, or engineers are allowed to access those deidentified analyses results through authorized internet connections to the study website. In conclusion, we believe that our data analytic approach to telemonitoring circadian and sleep cycles provides a new paradigm for the quantitative assessment of health risks in individual shift workers.

## Contributors

PG, FL, EC, DL and BF designed the collection of data from diaries and questionnaires, and FL, BFF, QH, SK, and AMA designed the data collection for the PiCADo platform. PG, FL, RA, GA and AMA organised the logistics for recruiting and telemonitoring health professionals volunteers. EC and PG organised and maintained the data base and advised on the use of the explanatory variables. DL and BF performed sleep debt analyses and advised on the interpretation of sleep scores. JAB, BFF, FL conceived the current project, designed the analysis pipeline for this work and supervised YZ in carrying out the analysis and synthesising the statistical results. YZ, JAB, BFF, FL interpreted this study and wrote the manuscript. SK advised on aspects of study design, and, together with AMA and QH, performed data collection and curation, and preliminary analyses of the PiCADo time series. YZ, EC, SK, QH, JAB, BFF and FL verified the underlying data. All authors contributed to the article and approved the submitted version.

## Data sharing statement

The input data to the cluster and regression analyses and R-codes to obtain results are available at (https://github.com/YiyuanZhang1995/Template-for-the-statistical-analysis-of-data-sets-including-activity-and-temperature). Deidentified participant questionnaire and telemonitored data will be made available to requesters after the date of publication, with no end date to availability. Data will be shared after an appropriate proposal is submitted and can be used for any purpose. Proposals should be directed to pascal.guenel@inserm.fr. Requests must be justified and will be subject to data access agreement between the parties.

## Declaration of interests

The authors declare that the research was conducted in the absence of any commercial or financial relationships that could be construed as a potential conflict of interest.

## Appendix Supplementary materials

Image, application 1
