# Supplementary material for: Digital circadian and sleep health in individual hospital shift workers: A cross sectional telemonitoring study
Source: eBioMedicine. 2022 Jun 27;81:104121. doi: 10.1016/j.ebiom.2022.104121 (PMC9253495; doi:10.1016/j.ebiom.2022.104121)
Supplement: Supplementary file 1 [file mmc1.docx]

Supplementary Materials for

**Digital circadian and sleep health in individual hospital shift workers: a cross sectional telemonitoring study**

Yiyuan Zhang, Emilie Cordina-Duverger, Sandra Komarzynski, Amal M Attari, Qi Huang, Guillen Aristizabal, Brice Faraut, Damien Léger, René Adam, Pascal Guénel, Julia A Brettschneider, Bärbel F Finkenstädt, Francis Lévi*

*Corresponding author. Email: [francis.levi@inserm.fr](mailto:francis.levi@inserm.fr)

Contents

[Supplementary Text 3](#_Toc93434572)

[1 Details of study design, ethics and conduct, inclusion criteria and data collection 3](#_Toc93434573)

[2 Models and Circadian Parameters 4](#_Toc93434574)

[3 Correlations between parameters on work and free days 7](#_Toc93434575)

[4 Relations between objective measures and parameters and subjective reports 8](#_Toc93434576)

[5 Cluster analysis 9](#_Toc93434577)

[6 Bivariate copula additive models 10](#_Toc93434578)

[Supplementary Figures 12](#_Toc93434579)

[Supplementary Tables 22](#_Toc93434580)

[Supplementary References 33](#_Toc93434581)

Supplementary Text

1 Details of study design, ethics and conduct, inclusion criteria and data collection

The *Circadiem* study aimed at the identification of circadian and sleep disorders as a function of shift work schedule in nurses and other health professionals working in the same public hospital. The study was sponsored by the Institut National de la Santé et de la Recherche Médicale (File #C15-66, INSERM, France). The protocol was approved by the “Comité consultatif sur le traitement de l’information en matière de recherche dans le domaine de la santé (File #16.153, approval granted on 23/03/2016), by the Ethical Committee of Ile-de France 1 (on 09/05/2016), and by the Commission Nationale Informatique et Libertés (January 2017). Recruitment was advertised through protocol presentations by the study coordinator at meetings that took place in each service (i.e. surgery, oncology, geriatrics, psychiatry, biochemistry, etc.) within Paul-Brousse Hospital, Villejuif, France. This hospital is part of the Assistance Publique - Hôpitaux de Paris (AP-HP), the largest French public hospital institution.

Participants were female or male nurses or other health professionals who worked on (i) day shifts consisting of morning shifts (from 7:00 to 14:00), alternating with afternoon shifts (from 14:00 to 21:00), (ii) night shifts (from 21:00 to 7:00), or (iii) ‘other’ shift schedules, involving different start and end times from one day to the next. They were instructed in detail about their expected tasks throughout the 6 to 11-day study period involving both work and free days and were included in the study after signing an informed consent form. Immediately following their inclusion into the study, they were asked to fill out a detailed sleep questionnaire, the French translation of the morningness-eveningness questionnaire (1,2), and questionnaires about lifestyle, including alcohol consumption, smoking, and coffee intake, as well as about socio-familial environment, including cohabitation and the number of children at home. Sleep debt (3) defined as self-reported ideal total sleep time minus total sleep time (both values were in the sleep questionnaire) was calculated.

To objectively determine the occurrence of circadian disruption and to characterize its dynamics, participants underwent telemonitoring of their activity (PA) and their chest surface temperature (Chesttemp) by means of a thoracic sensor (Movisens, Karlsruhe, Germany) worn on their anterior chest within a pocket located inside a dedicated vest or bra. The participants were asked to follow their usual daily routines throughout the study session. They were allowed to remove the chest sensor for up to 40 minutes daily throughout the study period. Whenever the sensor was not worn, chest surface temperature dropped conspicuously to the level of the environmental temperature, hence it was possible to reliably identify the corresponding temperature and activity data as missing values (4).

2 Models and Circadian Parameters

**2.1 Hidden Markov Modeling (HMM)**

Let $O_{t}$ represent the observation at time $t$ and $O^{\left( t \right)}=\{O_{1},O_{2},\ldots, O_{t}\}$ represent the history of observations from time 1 to time $t$, where $t\in\{1,2,\ldots,T\}$ and $T$ is the sample size. Let $S_{t}\in\{1,2,\ldots,m\}$ be the unobserved discrete state at time $t$, where $m$ is an integer denoting the number of states, and let $S^{(t)}=\{S_{1},S_{2},\ldots, S_{t}\}$ be the history of unobserved states from time 1 to $t$. The HMM consists of two parts (5):

(1) Unobserved state process: The sequence of states $S_{t}$ is a Markov chain satisfying the Markov property:

$$\Pr\left( S_{t} \right|S^{(t-1)})=\Pr\left( S_{t} \right|S_{t-1})$$

(2) State-dependent observation process: The $O_{t}$'s are independent conditional on $S_{t}$, and $O_{t}$ only depends on $S_{t}$:

$$\Pr\left( O_{t} \right|O^{\left( t-1 \right)}, S^{\left( t \right)})=\Pr\left( O_{t} \right|S_{t})$$

The joint distribution of the unobserved states and observations can be calculated by:

$$Pr(O^{\left( T \right)}, S^{\left( T \right)})=\Pr(S_{1})\prod_{t=2}^{T} \Pr\left( S_{t} \right|S_{t-1})\prod_{t=1}^{T} \Pr\left( O_{t} \right|S_{t})$$

The sum over the possible combination of states gives the probability of observations (5):

$$\Pr\left( O^{\left( T \right)} \right)=\sum_{S_{1}, S_{2},\ldots=1}^{M} \Pr(S_{1})\prod_{t=2}^{T} \Pr\left( S_{t} \right|S_{t-1})\prod_{t=1}^{T} \Pr\left( O_{t} \right|S_{t})$$

$$=\boldsymbol{\delta P}\left( O_{1} \right)\boldsymbol{\Gamma}\boldsymbol{P}\left( O_{2} \right)\boldsymbol{\Gamma}\ldots\boldsymbol{\Gamma}\boldsymbol{P}\left( O_{T} \right)\boldsymbol{1}$$

in which $\boldsymbol{\delta}$ $\in\mathbb{R}^{\boldsymbol{1\times m}}$ is the initial distribution of states (the distribution of $S_{1}$); $\boldsymbol{\Gamma}\in\mathbb{R}^{\boldsymbol{m\times m}}$is the transition probability matrix of Markov chain with entries $\boldsymbol{\Gamma}_{ij}= \Pr\left( S_{t}=j \right|S_{t-1}=i)$ and rows adding up to 1; $\boldsymbol{P}\left( O_{t} \right)\in\mathbb{R}^{\boldsymbol{m\times m}}$ is the conditional emission density matrix, which is a diagonal matrix with $j$'th diagonal element equal to $\Pr\left( O_{t} \right|S_{t}=j)$;$\boldsymbol{1}$ $\in\mathbb{R}^{\boldsymbol{m\times1}}$ is a column vector of ones. Consequently, the HMM is parametrized by the non-zero entries in unknown $\boldsymbol{\delta}\boldsymbol{,}\boldsymbol{\Gamma,}$and $\boldsymbol{P}\left( O_{t} \right)$ that need to be estimated. Given the observation $O^{\left( T \right)}$, the maximum-likelihood estimator of these unknown parameters can be efficiently found based on the Baum–Welch algorithm, for which closed-form expressions and computationally fast steps exist when the conditional emission density is Gaussian (5). The R package depmixS4 encodes the Baum–Welch algorithm and is used to fit HMM in this study. As the likelihood may have local maxima, ten different starting values are tested using the set.seed() command in R and comparison of model fits is based on Akaike's Information Criterion (AIC).

Define the vector $\boldsymbol{\alpha}_{\boldsymbol{t}}$ and $\boldsymbol{\beta}_{\boldsymbol{t}}^{\boldsymbol{'}}$, for $t\in\{1,\ldots,T\}$ as:

$$\boldsymbol{\alpha}_{\boldsymbol{t}}=\boldsymbol{\delta}\boldsymbol{P}\left( O_{1} \right)\boldsymbol{\Gamma}\boldsymbol{P}\left( O_{2} \right)\boldsymbol{\Gamma}\ldots\boldsymbol{\Gamma}\boldsymbol{P}\left( O_{t} \right)=\boldsymbol{\delta}\boldsymbol{P}\left( O_{1} \right)\prod_{k=2}^{t} \left( \boldsymbol{\Gamma}\boldsymbol{P}\left( O_{k} \right) \right)$$

$$\boldsymbol{\beta}_{\boldsymbol{t}}^{\boldsymbol{'}}=\boldsymbol{\Gamma}\boldsymbol{P}\left( O_{t+1} \right)\boldsymbol{\Gamma}\ldots\boldsymbol{\Gamma}\boldsymbol{P}\left( O_{T} \right)\boldsymbol{1}=\left( \prod_{k=t+1}^{T} \left( \boldsymbol{\Gamma}\boldsymbol{P}\left( O_{k} \right) \right) \right)\boldsymbol{1}$$

The elements of $\boldsymbol{\alpha}_{\boldsymbol{t}}$ are termed as forward probability and the $j$ 'th element $\alpha_{t}\left( j \right)$ is the joint probability of $\Pr\left( O_{1}=o_{1}, O_{2}=o_{2},\ldots, O_{t}=o_{t}, S_{t}=j \right)$. The elements of $\boldsymbol{\beta}_{\boldsymbol{t}}^{\boldsymbol{'}}$ are termed as backward probability and the $j$'th element $\beta_{t}\left( j \right)$ is the joint probability of $\Pr\left( O_{t+1}=o_{t+1}, O_{t+2}=o_{t+2},\ldots, O_{T}=o_{T} \right|S_{t}=j)$. It will then follow that, for $t\in\left\{ 2,\ldots,T \right\}$,

$\alpha_{t}(j)\beta_{t}(j)=\Pr\left. \left( O^{(T)}, S_{t}=j \right. \right)$ and $\boldsymbol{\alpha}_{\boldsymbol{t}}\boldsymbol{\beta}_{\boldsymbol{t}}^{\boldsymbol{'}}=\Pr\left. \left( O^{(T)} \right. \right)$. The predicted state at time $t$ can be estimated by (5):

$$\hat{S}_{t} = {argmax}_{\left\{ j=1, \ldots,m \right\}}\Pr(S_{t}=j| O^{\left( T \right)})= {argmax}_{\left\{ j=1, \ldots,m \right\}}\frac{\alpha_{t}(j)\beta_{t}(j)}{\Pr\left( O^{\left( T \right)} \right)}$$

This approach to determine the most likely state separately for each $t$ by maximising the conditional probability is called ''local decoding''.

***Harmonic HMM (HHMM)***: Considering the circadian timing system (CTS) in humans, it is natural to assume that the transition matrix in the Markov process follows a circadian cycle. We define the *harmonic HMM* (6), HHMM, where the $m$ hidden states are considered as a multinomial variable, so that the probabilities associated with every possible outcome can be estimated using multinomial logistic link function given a set of independent variables $t$. In this study, the logistic link function between $\boldsymbol{\Gamma}$ and $C_{t}$is given by (7):

$$\Pr\left. \left( S_{t}=k | S_{t-1}=j, C_{t} \right. \right)=\frac{exp\left( \beta_{j,k}^{0}+\beta_{j,k}^{1}C_{t} \right)}{\sum_{h=1}^{m} exp\left( \beta_{j,h}^{0}+\beta_{j,h}^{1}C_{t} \right)}$$

where $C_{t}$ is a simple cosinoid function with circadian period, i.e. $C_{t}= a*sin\left( \frac{2\pi t}{24} \right)+b*cos\left( \frac{2\pi t}{24} \right)$. For identification, one of each of the coefficients $\beta_{j,k}^{0}$ and $\beta_{j,k}^{1}$ are fixed for each row $j$, by setting $\beta_{j,j}^{0}=0$ and $\beta_{j,j}^{1}=0$.

***Two-oscillator HHMM (2-HHMM):*** Furthermore, assuming that transition probabilities on workdays and free days for the same subject are different, we invoke two oscillators as part of the HHMM, one for workdays and one for free days, i.e.

$${\begin{matrix} C_{t}=I_{t\in work}\left( a_{1}*sin\left( \frac{2\pi t}{24} \right)+b_{1}*cos\left( \frac{2\pi t}{24} \right) \right)+ \\ (1-I_{t\in work})\left( a_{2}*sin\left( \frac{2\pi t}{24} \right)+b_{2}*cos\left( \frac{2\pi t}{24} \right) \right) \end{matrix}}$$

where $I_{t\in work}$ is an indicator function that has a value of 1 when $t$ belongs to work days and a value of 0 otherwise.

**2.2 Definition of the four Circadian Parameters**

The first entry of transition matrix $\Pr\left( S_{t}=1 \right|S_{t-1}=1)$ or $\boldsymbol{\Gamma}_{11}$, the self-transition probability of the IA state, is denoted as p1-1 referring to the probability of staying in the IA state given that the previous state is the IA state. The value of p1-1 thus indicates the possibility of "staying in rest". As large values of p1-1 could indicate that a subject is unlikely to be interrupted during rest p1-1 can be used as an indicator of rest quality, i.e. high values of p1-1 are indicative of a high quality of rest. Note that in the HHMM the transition matrix $\boldsymbol{\Gamma}$ and thus $\boldsymbol{\Gamma}_{11}$ is subject to a 24h cycle leading with a total of 288 (=24h/5min) different values. We thus compute on overall weighted average value of p1-1 where the weights are given by the probability of rest at the corresponding time point over the sum of probability of rest of every time point:

$$weighted p1­1=\sum_{t=1}^{N} p1­1_{t}*\frac{p1_{t}}{\sum_{h=1}^{N} p1_{h}}$$

where $p1­1_{t}$ is the estimated p1-1 at time $t$; $p1_{t}$ is the estimated probability of rest at time $t$; $N$ is total time point in a day, which in this case is 288; the last fraction term works as the weight. Similar to p1-1, the weighted p1-1 indicates the rest quality. The difference between p1-1 by HMM and the weighted p1-1 in HHMM is that HMM tries to do an average of activity over the whole study period, while the harmonic HMM gives more weights to the data by which the subject is estimated to be in rest state.

The second circadian parameter is RI, which refers to ***Rhythm Index*** created by (6):

$RI=\frac{24}{24-a}* \left. \left( \frac{1}{a}\int_{t\in I_{c}} \Pr\left( S_{t}=1 \right)dt-\frac{a}{24} \right. \right)$.

In this equation, $a=\int_{t} \Pr\left( S_{t}=1 \right)dt$ is the area of the blue part in **Fig. S2** and represents the rest amount (in hours), $c$ is the gravity centre of the blue part and represents the centre time of rest (clock time), $I_{c}= \left[ c-\frac{a}{2},c+\frac{a}{2} \right]$. Rest amount and centre time of rest are the remaining two circadian parameters. Consider an example given in **Fig. S2** where the subject has a total of 7·7 hour of rest ($a$=7·7) and the centre time of rest occurs at 03:18 am ($c$=3·3). Given the area and the centre of the blue part representing the amount of rest an individual had, the light and dark grey rectangles have the same area and centre than the blue part but they represent two extreme cases in which the individual’s probability of rest could be distributed over the circadian cycle for the same rest duration and centre. In the scenario represented by the light grey rectangle, the probability of rest is equal to 1, i.e. there is no interruption, and the same rest amount $a$ distributed around the same center $c$ but with a completely regular onset and offset of the (monophasic) rest. On the other hand absence of circadian rhythm is indicated by the dark grey rectangle where the probability of rest is distributed equally over the course of the day and is equal to $\frac{a}{24}$ . The profile of a normal rest for subjects would lie between the best and worst scenario such as for example the blue area in **Fig. S2**. RI is constructed to compare the rest profile with the best and worst case. From the Equation of RI, the maximum value of RI is 1 under the condition that $\int_{t\in I_{c}} \Pr\left( S_{t}=1 \right)dt=a$, as the light grey rectangle. The minimum value of RI is 0 when $\int_{t\in I_{c}} \Pr\left( S_{t}=1 \right)dt=\frac{a^{2}}{24}$, as the dark grey rectangle. However, for a given centre $c$ and duration $a$ of rest a value of RI close to one could indicate that an individual has enjoyed a regular recurrence and high quality of rest. Note that the RI is based on the personal values of $a$ and $c$ but in itself does not evaluate the duration $a$ and center $c$ of rest which are however estimated and available as parameters from the HHMM. These are considered as further circadian parameters in our analysis.

3 Correlations between parameters on work and free days

For both DS and NS groups, Spearman correlations supported consistency of individual circadian PA parameter values on work and free days for p1-1 (DS: r=0·4356, p<0·0001; NS: r=0·3383, p=0·0069), rest amount (DS: r=0·2032, p=0·076; NS: r=0·4664, p=0·0001) and rest centre time (DS: r=0·2377, p=0·0373; NS: r=0·2540, p=0·0446), thus indicating that subjects with higher quality and quantity of rest on work days were likely to have these also on free days. While the DS also had significantly correlated RI’s on work and free days (r=0·2817, p=0·013), this was not the case for the NS (r=0·0749, p=0·56), because of their low RI values on work days and partial recovery on free days.

4 Relations between objective measures and parameters and subjective reports

The HMM-estimated rest amounts from PA data were positively correlated on work and free days, with (i) the subjective nocturnal sleep duration from questionnaire (workdays: Spearman correlation r=0·2624, p=0·0017; free days: r=0·1925, p=0·023) and (ii) the self-reported sleep duration from diary (workdays: r=0·3900, p<0·0001; free days: r=0·1148, p=0·18). However, HMM-estimated rest amount from rest-activity time series tended to be longer than sleep duration computed from questionnaire or diary (p<0·0010 for both work and free days). The average differences between rest amount and sleep duration on workdays were 1·5 and 2·7 h (rest amount minus sleep duration from questionnaire) for DS and NS, and 1·6 and 2·6 h (rest amount minus sleep duration from diary) for DS and NS, respectively. Such differences between objective and subjective measures of rest were less than 1 h on free days, both for DS and NS. Similarly, the centre time of rest, as estimated by 2-HHMM, occurred earlier than that from diary on workdays (-1·8h for DS; -2·7h for NS), whilst minimal differences, if any, were found on free days (-0·4h for DS; 0·0h for NS). The HMM-estimated centre time of rest was highly correlated with the reported mid-time of sleep in diary both on work days (r=0·7635, p<0·0001 for all subjects; r=0·3564, p=0·0017 for DS; r=0·4636, p=0·0001 for NS) and on free days (r=0·6867, p<0·0001 for all subjects; r=0·5477, p<0·0001 for DS; r=0·7791, p<0·0001 for NS).

5 Cluster analysis

**5.1 Details of performing cluster analysis**

To perform cluster analysis, the rest profiles for work and free days, i.e. the curves given by the probability of being in the rest state over the course of a day, estimated by the 2-HHMM, were concatenated into one vector which contained 576 elements for each participant, i.e. 288 entries for each rest profile evaluated at 5-minute intervals from 20:00 to 19:59 with data extracted from either just work or free days. The starting time of 20:00 was chosen to keep nights within the same period and following, roughly, the existing workdays of NS (as detailed in section 2·1 in the manuscript, NS workdays were from 20:00 to 19:59 or from 21:00 to 20:59). As DS workdays technically range from 0:00 to 23:59, the data was divided into two parts, one from 0:00 to 19:59 and the other from 20:00 to 23:59, and slotted in accordingly. (Technically speaking, the 0:00 to 19:59 component was placed after the interval from 20:00 to 23:59 to generate data over the interval from 20:00 to 19:59.)

The **Fig. S4** shows that the within-cluster sum of square flattens out after 2 or 3 clusters for DS (yellow line) and NS (blue line). We decided to use 3 clusters for both shift groups as introducing a third cluster provided a further meaningful separation of a very large cluster under the 2-cluster scenario.

**5.2 Additional results in the comparison of clusters**

Beside the differences in circadian parameters among clusters, chronotype scores were lowest thus supporting more eveningness in DS cluster 3 (p=0·0454) as well as in NS cluster 3 (p=0·0012) (**Tables S3a** and **S3b**). There were more cohabitants for DS and NS in clusters 1. No significant differences in age, BMI or sleep debt were found between the three DS or the three NS clusters. There was a trend toward a larger BMI and more years of night shift work for the NS in cluster 2. For DS, the Chesttemp spectra only peaked at 24h for cluster 1, but at both 24h and 12h for clusters 2 and 3 (**Fig. 5m**). Thus, a higher proportion of DS in cluster 1 had a 24h temperature period (top panel in **Fig. 5n**). For NS, the dominant Chesttemp periods were similar for all three clusters (**Fig. 5p**), although subjects in cluster 2 had a marked spectral peak at 8h (**Fig. 5o**). The height of the 12h and 24h peaks were similar in cluster 3 indicating a strong prevalence of the shorter 12h cyclicity while NS in clusters 1 and 2 were more subject to 24h periodicity in their Chestemp. The detailed pairwise comparisons between any two DS or NS clusters are summarised in **Tables** **S4a** and **S4b**, respectively.

6 Bivariate copula additive models

For model building, a normal Q–Q plot of quantile residuals for each margin was used to determine the fitness of the marginal distributions (log odds of p1-1 on work and free days: the Gaussian distribution; log odds of RI on work and free days: the Gaussian distribution; rest amount on work and free days: the Weibull distribution; rest centre time on work and free days: the Weibull distribution and the Gaussian distribution respectively). The corrected Akaike Information criterion AICc (13) was applied to choose a copula function. The model including all candidate variables with selected marginal distributions and copula function was used as starting point for variable selection. A backward elimination using stepwise regression was applied involving an automatic model selection procedure (14) with model evaluation based on AICc. Recall that information criteria such as the AIC strike a trade-off between the [goodness of fit](https://en.wikipedia.org/wiki/Goodness_of_fit) and the parsimony of the model penalising the likelihood by a term that includes the number of parameters. AICc also accounts for small sample size. The backward elimination involved testing the relevance of variables by deleting at each step the variable (if any) whose loss gives the most statistically insignificant deterioration of the model fit, here according to the AICc, and then repeating this process until the AICc no longer decreases. The link functions were selected to meet the requirements of the parameter spaces (log odds of p1-1 on work and free days: the identity function; log odds of RI on work and free days: the identity function; rest amount on work and free days: $log\left( \cdot- \epsilon\right)$ with $\epsilon=1e07$; rest centre time on work and free days: $log\left( \cdot- \epsilon\right)$ and the identity function respectively). After the model with the most relevant covariates was built on the whole data set, Cook’s distance (15) was calculated to investigate the influence of each observation on the fitted response values. Any observations with Cook’s distance larger than $\frac{4}{n}, n$ is the sample size, were considered as outliers. Finally, a new model was built on the dataset without outliers and was used to investigate the effect of covariates and their interactions on the four circadian parameters, each taken as response variable in the regression model. The R package GJRM was used to fit bivariate copula additive models in this study. We generally report the significance of a covariate effect by the p-value of the two-sided z-test.

Supplementary Figures


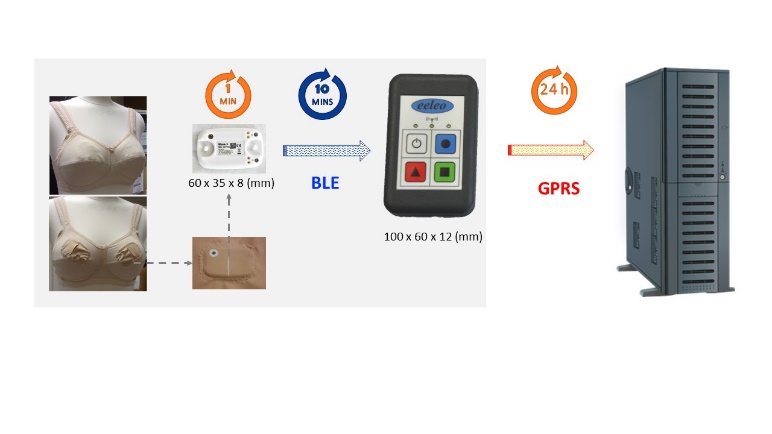


**Supplementary Figure S1. Illustration of PiCADo mobile e-Health platform**


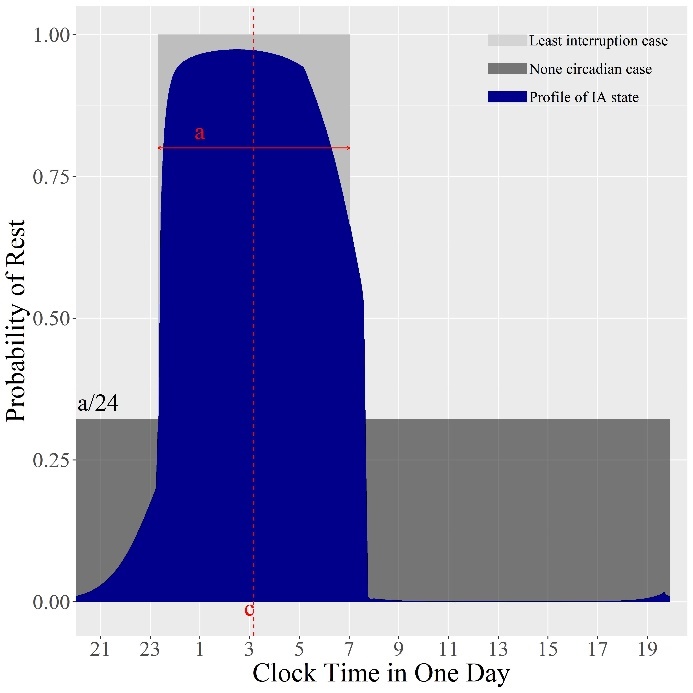


**Supplementary Figure S2. Definition of RI.** The top profile of the blue area is given by the probability of rest (or in the IA state) every 5 minutes which can be estimated per subject on the basis of the HHMM. The blue area represents the rest amount (hour) per day, or the total time per day spend in the IA state. The gravity centre c of the blue area is an estimator of the centre time of rest (clock time).


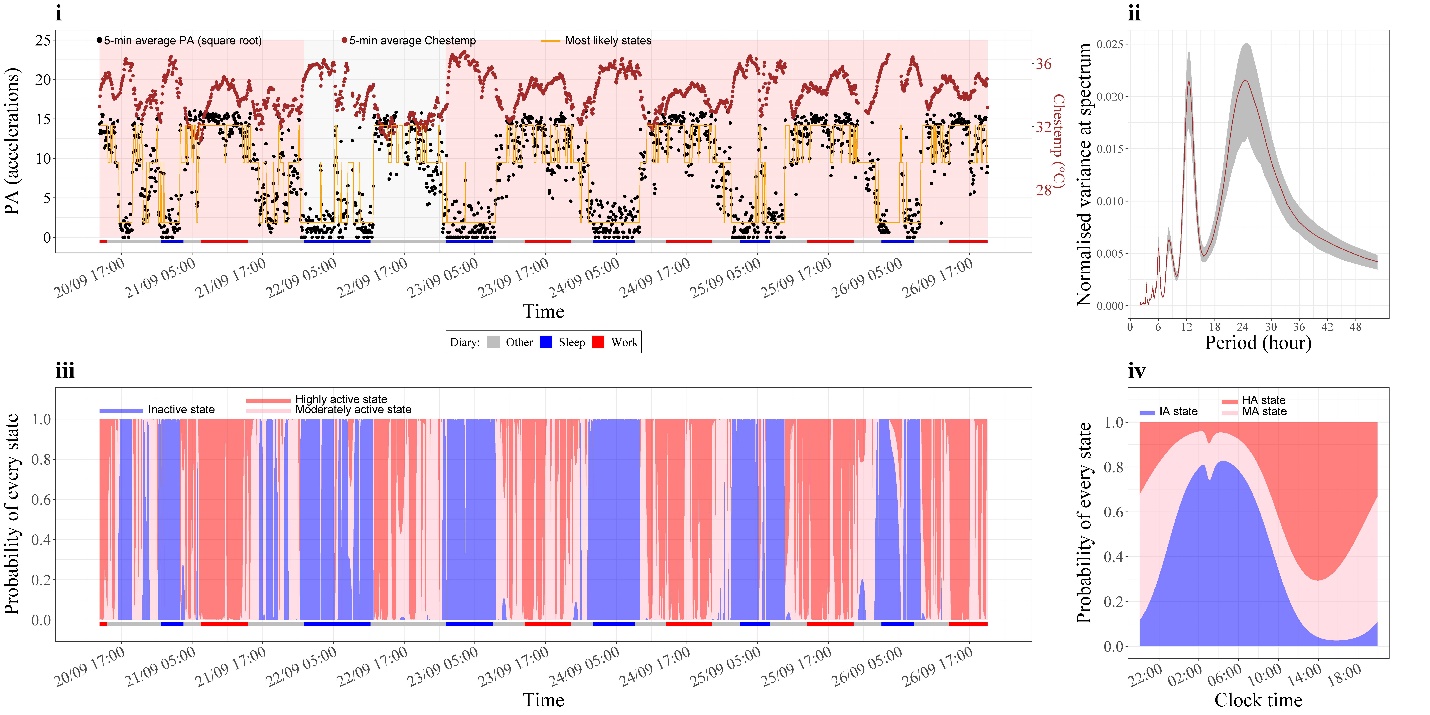


**Supplementary Figure S3a.** **Subject 1152 (52 y.o, female, DS)**


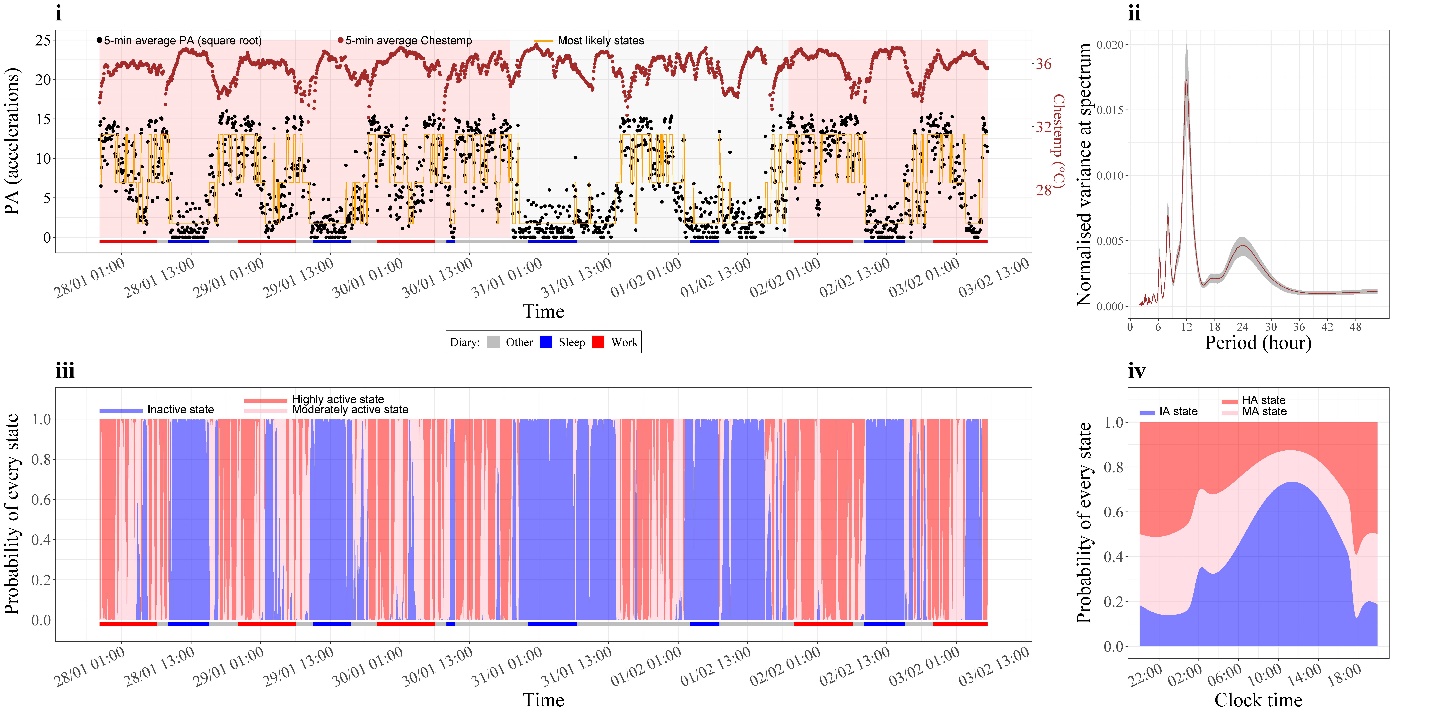


**Supplementary Figure S3b.** **Subject 1529 (38 y.o. female, NS).** (**i**) Time series of PA (black dots) and Chesttemp (brown dots) with yellow line indicating the most likely state using local decoding. The red and blue horizontal bars denote the work and sleep periods recorded in the diary. (**ii**) Spectral density estimates of Chesttemp (brown line) with respective 90% confidence intervals (grey area). (**iii**) State probability plot during the whole study period, i.e. cumulative plot of $Pr(S_{t}=j| O^{\left( T \right)})$for $j$ = 1 (IA, blue), 2 (MA, pink), 3 (HA, red). (**iv**) Circadian state probability plot from HHMM obtained as the averaged probability of every state over the whole study period.


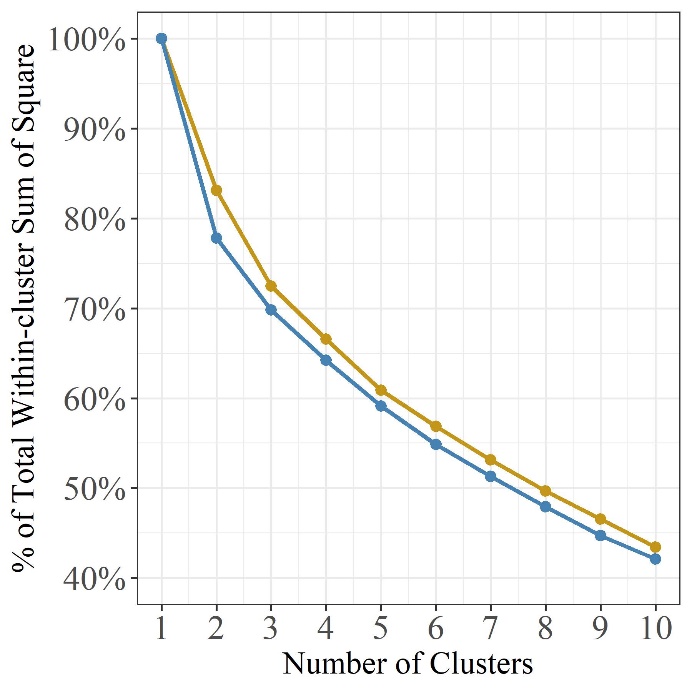


**Supplementary Figure S4.** **Plot of change in within-cluster sum of squares.** It looks like the within-cluster sum of square flattens out after 2 or 3 clusters for DS (yellow line) and NS (blue line). We decided to use 3 clusters for both shift groups as introducing a third cluster provided a further meaningful separation of a very large cluster under the 2-cluster scenario. Three clusters involved 34, 24, and 19 DS, and 28, 11, and 24 NS respectively.

**
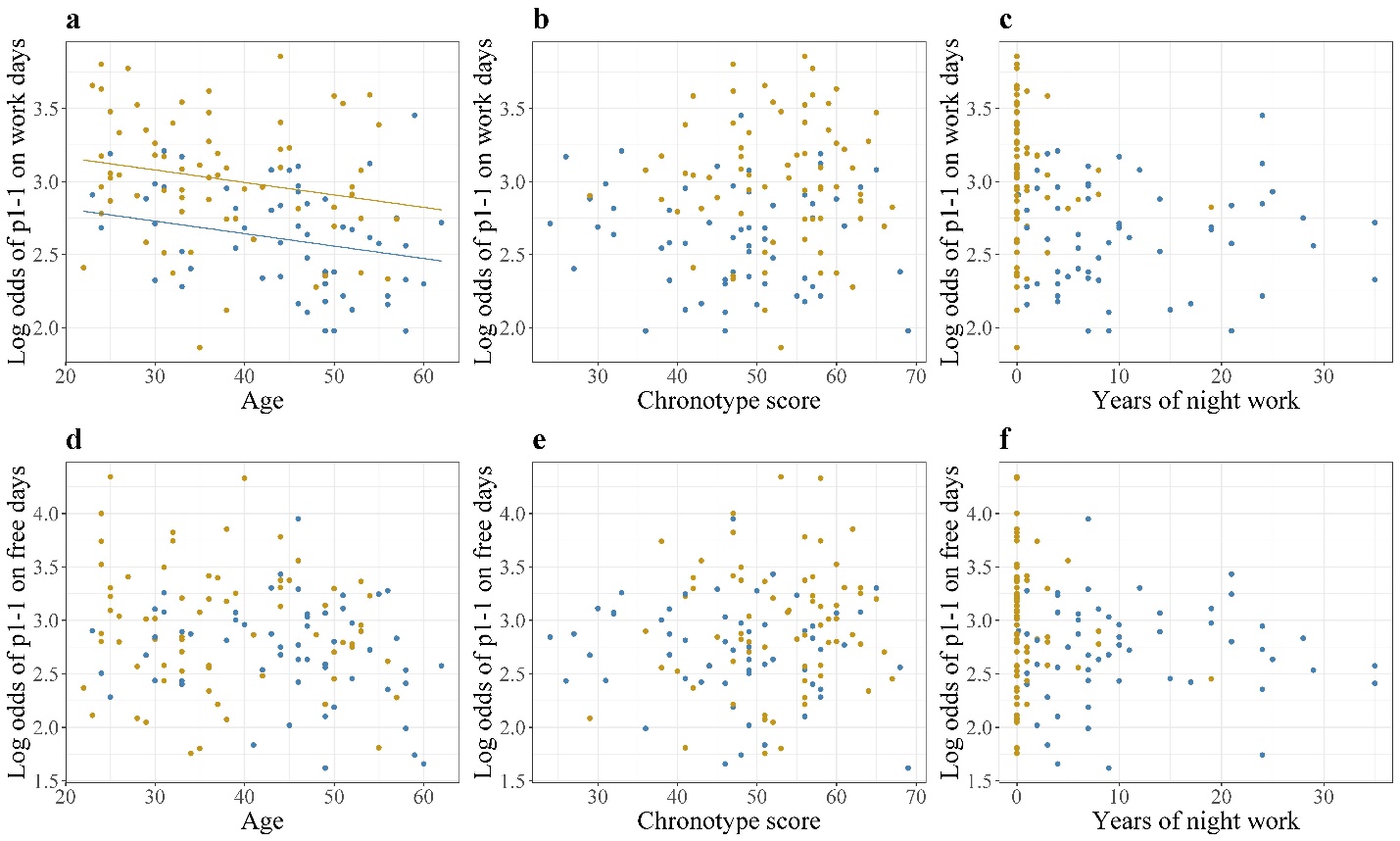
**

**Supplementary Figure S5.** **Scatterplots of log odds of p1-1 vs three covariates.** Left, middle and right columns show the relationship between log odds of p1-1 (LOP) and age, between LOP and chronotype score, and between LOP and years of night work respectively (yellow for DS, blue for NS). The estimated regression lines from the model for LOP are plotted if a covariate has significant effect on LOP (yellow for DS, blue for NS).

**
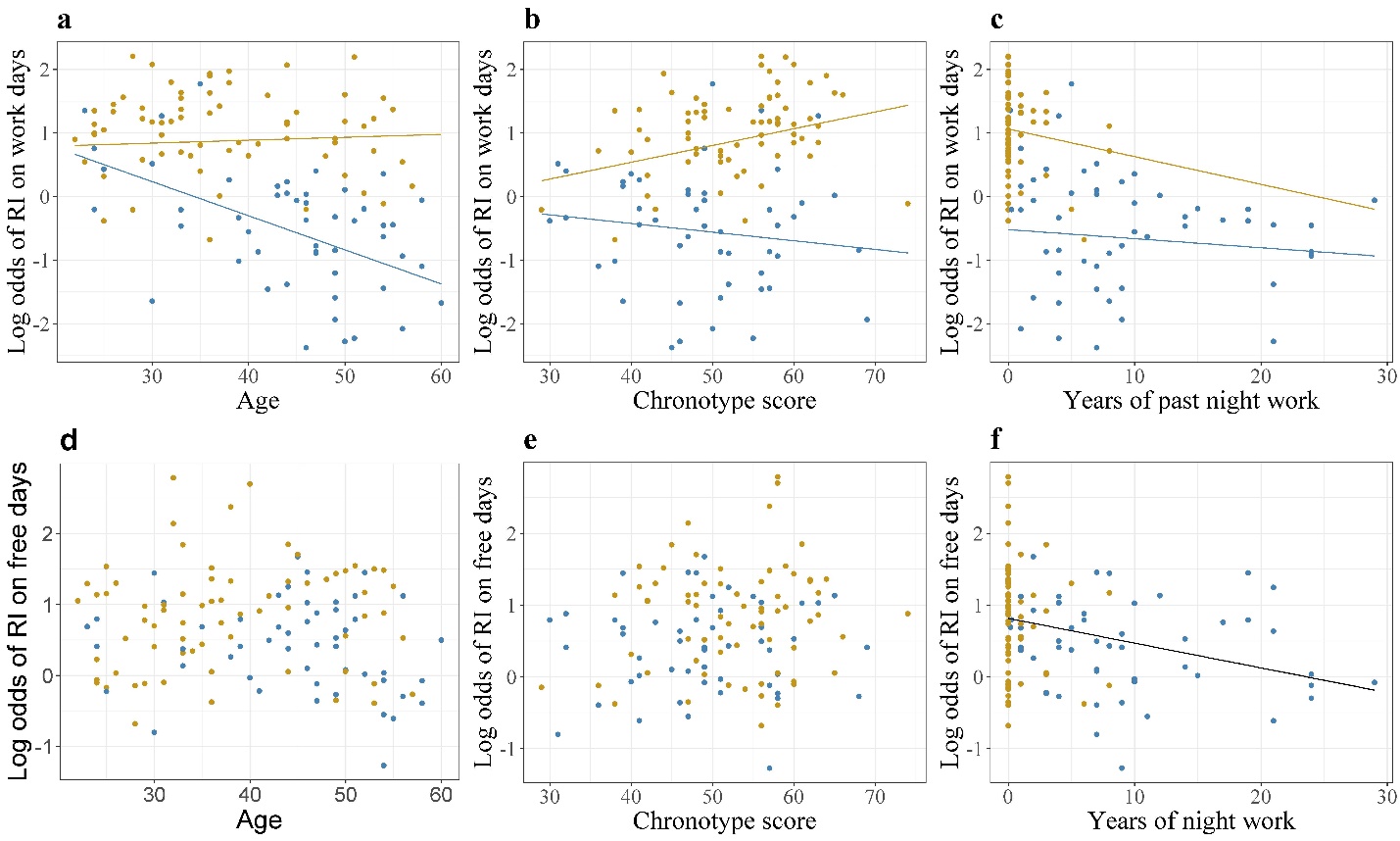
**

**Supplementary Figure S6.** **Scatterplots of log odds of RI vs three covariates.** Left, middle and right columns show the relationship between log odds of RI (LORI) and age, between LORI and chronotype score, and between LORI and years of night work respectively (yellow for DS, blue for NS). The estimated regression lines from the model for LORI are plotted if a covariate has significant effect on LORI. When the shift type plays a role in the model, two regression lines are given (yellow for DS, blue for NS). Otherwise, one regression line in black is given.

**
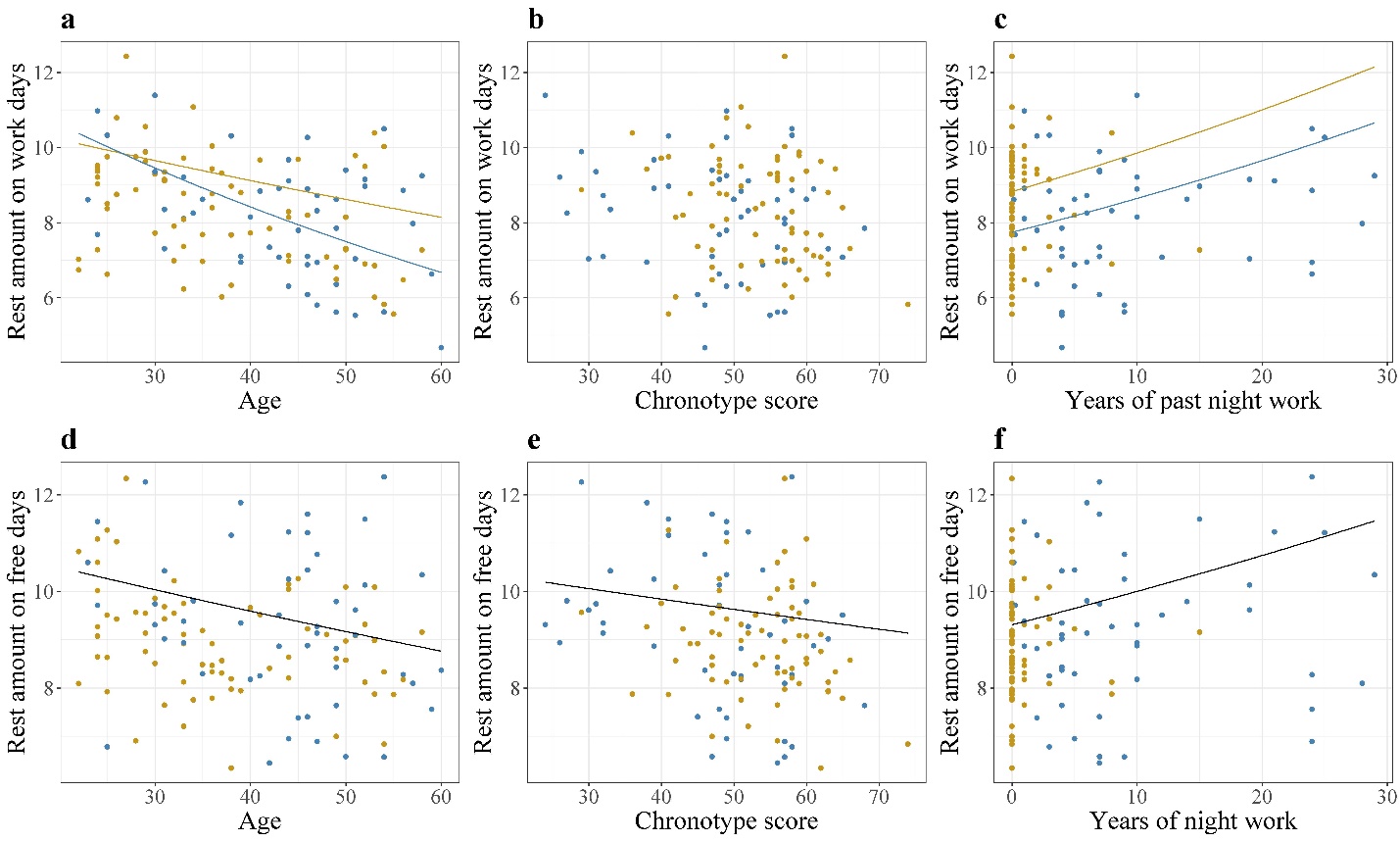
**

**Supplementary Figure S7.** **Scatterplots of rest amount vs three covariates.** Left, middle and right columns show the relationship between rest amount and age, between rest amount and chronotype score, and between rest amount and years of night work respectively (yellow for DS, blue for NS). The estimated regression lines from the model for rest amount are plotted if a covariate has significant effect on rest amount. When the shift type plays a role in the model, two regression lines are given (yellow for DS, blue for NS). Otherwise, one regression line in black is given. Note that due to the logarithm link function, regression lines may not be straight.


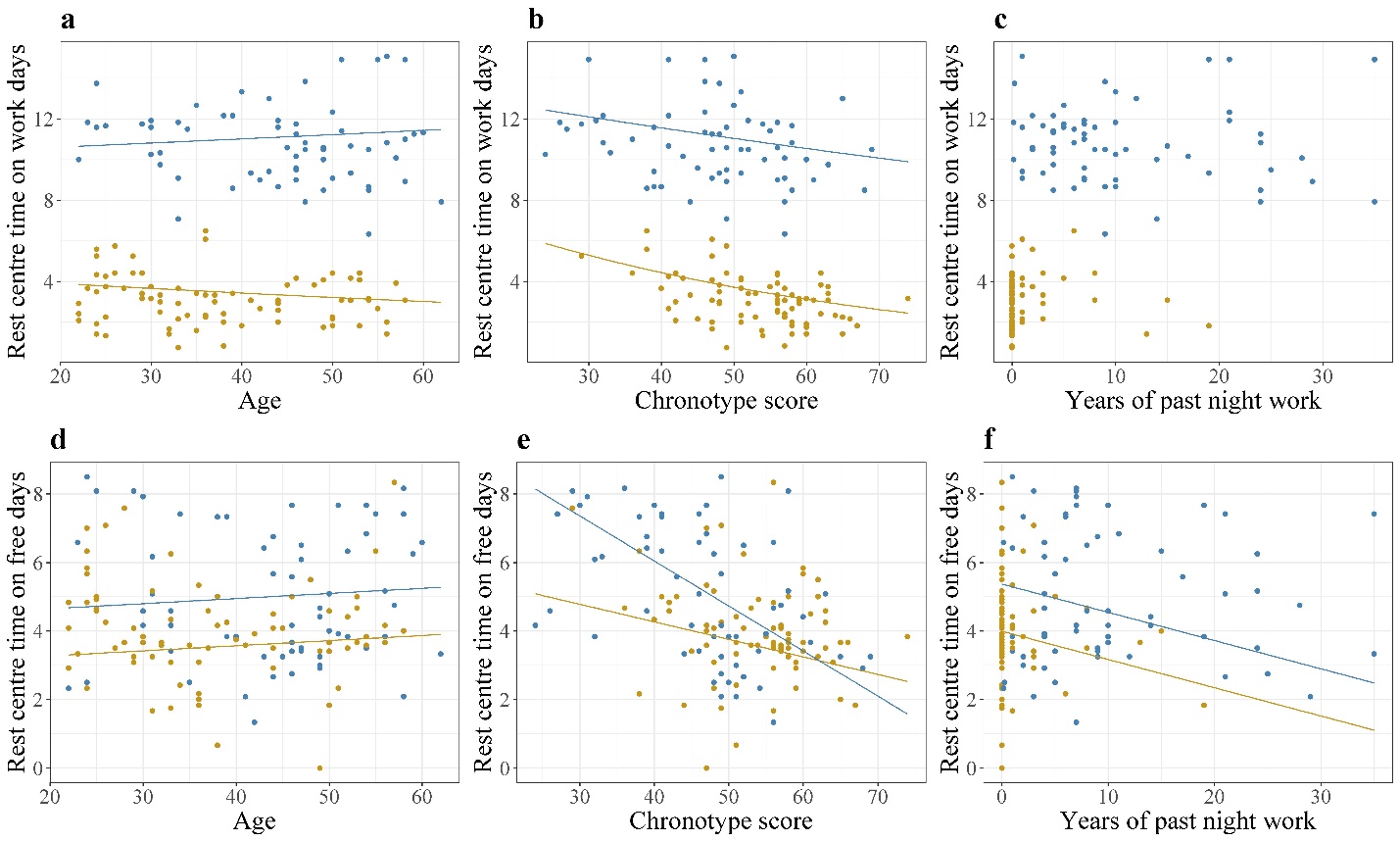


**Supplementary Figure S8.** **Scatterplots of rest centre time vs three covariates.** Left, middle and right columns show the relationship between rest centre time and age, between rest centre time and chronotype score, and between rest centre time and years of night work respectively (yellow for DS, blue for NS). The estimated regression lines from the model for rest centre time are plotted if a covariate has significant effect on rest amount. When the shift type plays a role in the model, two regression lines are given (yellow for DS, blue for NS). Note that due to the logarithm link function, regression lines may not be straight. One subject whose rest centre time on free days is 0:00 (in panel **e**), which is the reason of using identity link for $\mu_{free}^{CT}$ rather than $log\left( \cdot- \epsilon\right)$.


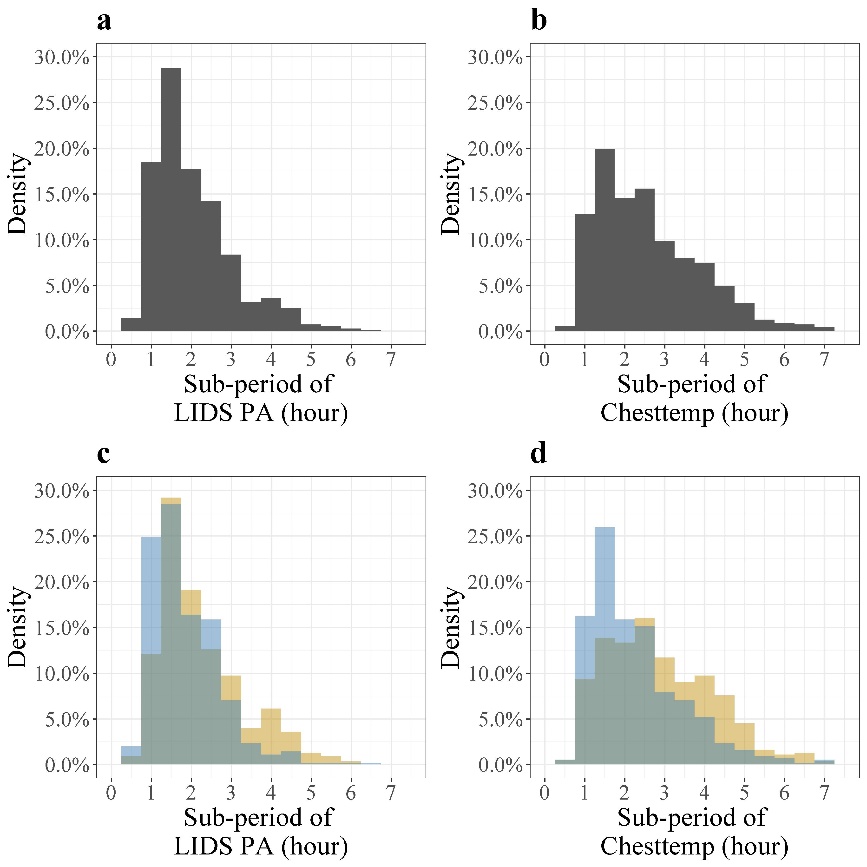


**Supplementary Figure S9. Sub-periods in LIDS PA and Chesttemp.** (**a-b**) Histograms of the distribution of sub-periods in Chesttemp and LIDS PA for all subjects. The median of sub-periods in Chesttemp is 2·3 [IQR, 1·6-3·3] and in LIDS PA is 1·7 [1·3-2·5]. (**c-d**) The distributions of sub-periods for DS and NS are plotted separately but overlay with each other (yellow for DS, blue for NS).


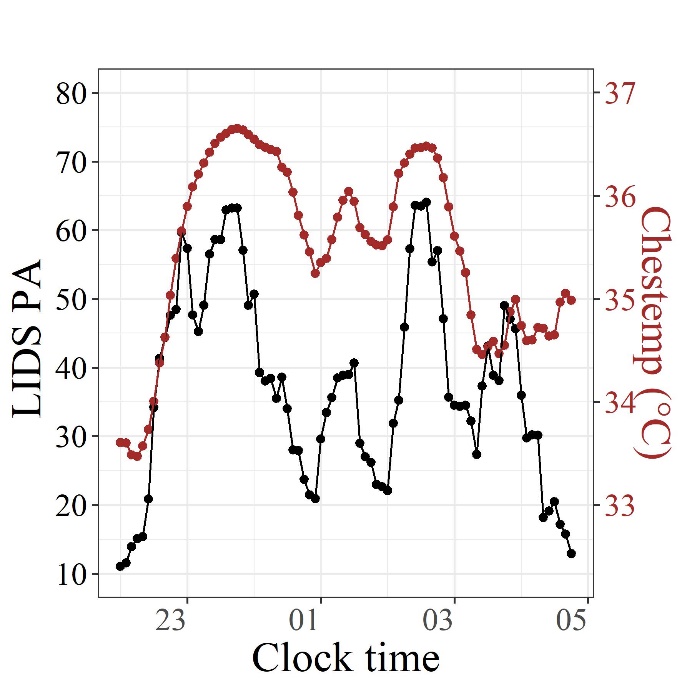


**Supplementary Figure S10. Example of LIDS PA and Chesttemp during one rest bout (subject 1101, 30 y.o, female, DS).**

Supplementary Tables

**Supplementary Table S1.** **Characteristics** **of the population in DS and NS included in the study.**

|  | **Shift groups** | | | | | | **p^(c)^** | |
| --- | --- | --- | --- | --- | --- | --- | --- | --- |
|  | **DS (n=77)** | | |  | **NS (n=63)** | |  |  |
| Socio-demographic characteristics |  | |  |  |  |  |  |  |
| Age |  | |  |  |  |  |  |  |
| mean (SD) | 37·5 | | 10·8 |  | 44·1 | 10·4 | 0·0008 | *** |
| Sex |  | |  |  |  |  |  |  |
| Women | 74 | | 96% |  | 59 | 94% | 0·79 |  |
| Men | 3 | | 4% |  | 4 | 6% |  |  |
| Cohabitant |  | |  |  |  |  |  |  |
| No | 26 | | 34% |  | 23 | 37% | 0·87 |  |
| Yes | 51 | | 66% |  | 40 | 63% |  |  |
| Number of children at home |  | |  |  |  |  |  |  |
| 0 | 26 | | 34% |  | 21 | 33% | 0·51 |  |
| 1 | 23 | | 30% |  | 15 | 24% |  |  |
| ≥ 2 | 28 | | 36% |  | 27 | 43% |  |  |
| Characteristics of work |  | |  |  |  |  |  |  |
| Position |  | |  |  |  |  |  |  |
| Care assistant | 39 | | 51% |  | 31 | 49% | 0·21 |  |
| Nurse | 32 | | 42% |  | 31 | 49% |  |  |
| Health executive | 6 | | 8% |  | 1 | 2% |  |  |
| Number of years in current position |  | |  |  |  |  |  |  |
| mean (SD) | 2·7 | | 3·6 |  | 9·4 | 9·0 | <0·0001 | *** |
| Number of hours worked per week |  | |  |  |  |  |  |  |
| mean (SD) | 35·3 | | 3·4 |  | 32·8 | 1·1 | <0·0001 | *** |
| Duration of travel time (hours) |  | |  |  |  |  |  |  |
| mean (SD) | 0·6 | | 0·4 |  | 0·7 | 0·6 | 0·40 |  |
| Day shift type^(b)^ |  | |  |  |  |  |  |  |
| Morning | 17 | | 22% |  |  |  |  |  |
| Mixed | 45 | | 58% |  |  |  |  |  |
| Afternoon | 15 | | 19% |  |  |  |  |  |
| Number of work or free days on study session |  | |  |  |  |  |  |  |
| Workdays | 4·3 | | 1·1 |  | 3·8 | 0·6 | 0·0084 | ** |
| Free days | 2·1 | | 0·7 |  | 2·4 | 0·6 | 0·0019 | ** |
| Night shift work history |  | |  |  |  |  |  |  |
| Number of years in night work (for ever night worker) ^(a)^ | |  |  |  |  |  |  |  |
| < 5 years | 14 | | 67% |  | 19 | 30% |  |  |
| 5-10 years | 4 | | 19% |  | 24 | 38% |  |  |
| >10 years | 3 | | 14% |  | 20 | 32% |  |  |
| mean (SD) | 4·8 | | 5·1 |  | 10·5 | 8·9 | 0·0006 | *** |
| Sleep |  | |  |  |  |  |  |  |
| Epsworth score |  | |  |  |  |  |  |  |
| No sleepiness | 42 | | 55% |  | 35 | 56% | 0·25 |  |
| Sleepiness | 27 | | 35% |  | 26 | 41% |  |  |
| Severe sleepiness | 8 | | 10% |  | 2 | 3% |  |  |
| mean (SD) | 10·3 | | 4·2 |  | 9·2 | 4·4 | 0·18 |  |
| Nocturnal sleep duration during workdays (h) ^(d)^ |  | |  |  |  |  |  |  |
| mean (SD) | 6·8 | | 1·3 |  | 5·0 | 1·8 | <0·0001 | *** |
| Nocturnal sleep duration during free days (h) ^(d)^ |  | |  |  |  |  |  |  |
| mean (SD) | 8·5 | | 1·5 |  | 8·3 | 1·8 | 0·37 |  |
| Sleep debt (h) |  | |  |  |  |  |  |  |
| mean (SD) | 1·0 | | 1·4 |  | 2·2 | 1·6 | 0·0002 | *** |
| Lifestyle habits |  | |  |  |  |  |  |  |
| Alcohol |  | |  |  |  |  |  |  |
| Never | 46 | | 60% |  | 33 | 52% | 0·68 |  |
| < 2 drinks/week | 17 | | 22% |  | 17 | 27% |  |  |
| ≥ 2 drinks/week | 14 | | 18% |  | 13 | 21% |  |  |
| Current consumption | 1·1 | | 2·2 |  | 0·9 | 1·6 | 0·58 |  |
| Tobacco |  | |  |  |  |  |  |  |
| Never smoker | 48 | | 62% |  | 34 | 54% | 0.24 |  |
| Former smoker | 8 | | 10% |  | 13 | 21% |  |  |
| Current smoker | 21 | | 27% |  | 16 | 25% |  |  |
| Current consumption (nb/d) | 10·2 | | 6·3 |  | 13·4 | 4·4 | 0·072 | . |
| Body Mass Index (BMI) |  | |  |  |  |  |  |  |
| Normal or lean (<25) | 43 | | 56% |  | 25 | 40% | 0·11 |  |
| Overweight (25-30) | 18 | | 23% |  | 24 | 28% |  |  |
| Obese (>30) | 16 | | 21% |  | 14 | 22% |  |  |
| mean (SD) | 25·4 | | 5·4 |  | 26·4 | 4·7 | 0·071 | . |
| Chronotype score |  | |  |  |  |  |  |  |
| Morning | 20 | | 26% |  | 6 | 10% | 0·0023 |  |
| Intermediate | 49 | | 64% |  | 38 | 61% |  | ** |
| Evening | 7 | | 9% |  | 18 | 29% |  |  |
| mean (SD) | 53·3 | | 8·3 |  | 47·3 | 10·4 | 0·0006 | *** |
| Menopause |  | |  |  |  |  |  |  |
| NA | 3 | |  |  | 4 |  |  |  |
| Post-menopause | 13 | | 18% |  | 26 | 44% | 0·0017 | ** |
| Pre-menopause | 61 | | 82% |  | 33 | 56% |  |  |
| Medical history |  | |  |  |  |  |  |  |
| Personal diseases |  | |  |  |  |  |  |  |
| Cardio-vascular diseases | 2 | | 3% |  | 4 | 6% | 0·50 |  |
| Metabolic diseases | 13 | | 17% |  | 11 | 17% | 1·00 |  |
| Digestive and renal diseases | 9 | | 12% |  | 12 | 19% | 0·33 |  |
| Neurological diseases | 20 | | 26% |  | 16 | 25% | 1·00 |  |
| Respiratory diseases | 9 | | 12% |  | 14 | 22% | 0·15 |  |
| Musculoskeletal diseases | 6 | | 8% |  | 6 | 10% | 0·95 |  |
| Cancers | 0 | | 0% |  | 2 | 3% | 0·39 |  |
| Medical Treatment |  | |  |  |  |  |  |  |
| Analgesic treatment | 11 | | 14% |  | 13 | 21% | 0·44 |  |
| Anti-inflammatory treatment | 6 | | 8% |  | 11 | 17% | 0·14 |  |
| Hormonal treatment | 6 | | 8% |  | 4 | 6% | 1·00 |  |
| Digestive treatment | 2 | | 3% |  | 7 | 11% | 0·090 | . |
| Anti-cholesterol treatment | 0 | | 0% |  | 3 | 5% | 0·18 |  |
| Antidiabetic treatment | 1 | | 1% |  | 4 | 6% | 0·25 |  |
| Antihypertensive treatment | 3 | | 4% |  | 8 | 13% | 0·11 |  |
| Cardiological treatment | 2 | | 3% |  | 3 | 5% | 0·88 |  |
| Anxiolytic or antidepressant treatment | 8 | | 10% |  | 2 | 3% | 0·19 |  |
| Sleep (from diary) ^(d)^ |  | |  |  |  |  |  |  |
| Sleep duration per 24h during workdays (h) | | |  |  |  |  |  |  |
| mean (SD) | 6·7 | | 1·3 |  | 5·2 | 1·5 | <0·0001 | *** |
| Sleep duration per 24h during free days (h) |  | |  |  |  |  |  |  |
| mean (SD) | 8·2 | | 1·2 |  | 8·6 | 1·5 | 0·039 | * |
| Sleep midpoint during workdays |  | |  |  |  |  |  |  |
| mean (SD) | 4·9 | | 2·2 |  | 11·9 | 1·6 | <0·0001 | *** |
| Sleep midpoint during free days |  | |  |  |  |  |  |  |
| mean (SD) | 4·4 | | 1·2 |  | 5·1 | 1·9 | 0·11 |  |

^(a)^ Missing data for 1 DS and 1 NS.

^(b)^ Morning or afternoon day shift type is determined by the work time in diary; mixed shift means monitoring on both morning and afternoon shifts.

^(c)^ Statistical comparisons between groups with Chi-square tests for categorical characteristics and Mann-Whitney U test for the other ones.

^(d)^The relations between objective measures and subjective reports from questionnaire and diary are provided in SM-4.

Significance codes: 0 ‘***’ 0·001 ‘**’ 0·01 ‘*’ 0·05 ‘.’ 0·1 ‘ ’ 1

**Supplementary** **Table S2a. Summary of four circadian parameters for DS and NS during the entire study session**

|  |  | DS | NS |
| --- | --- | --- | --- |
| p1-1 | mean | 0·9480 | 0·9371 |
|  | SD | 0·0449 | 0·0417 |
|  | median | 0·9573 | 0·9438 |
|  | IQR | [0·9449, 0·9665] | [0·9356, 0·9529] |
| RI | mean | 0·6755 | 0·3789 |
|  | SD | 0·1229 | 0·1198 |
|  | median | 0·6920 | 0·3750 |
|  | IQR | [0·6030, 0·7700] | [0·2925, 0·4655] |
| Rest Amount | mean | 8·7 | 8·2 |
|  | SD | 1·2 | 1·5 |
|  | median | 8·9 | 8·2 |
|  | IQR | [7·9, 9·5] | [7·3, 9·3] |
| Centre time | mean | 3·4 | 7·3 |
|  | SD | 1·1 | 1·9 |
|  | median | 3·3 | 6·9 |
|  | IQR | [2·8, 3·9] | [6·0, 8·9] |

**Supplementary** **Table S2b. Summary of four circadian parameters for DS and N7S on work and free days**

|  |  | DS | | NS | |
| --- | --- | --- | --- | --- | --- |
|  |  | Workdays | Free days | Workdays | Free days |
| p1-1 | mean | 0·9449 | 0·9419 | 0·9216 | 0·9228 |
|  | SD | 0·0322 | 0·0352 | 0·0474 | 0·0561 |
|  | median | 0·9536 | 0·9478 | 0·9331 | 0·9387 |
|  | IQR | [0·9396, 0·9536] | [0·9281, 0·9665] | [0·9098, 0·9475] | [0·9181, 0·9546] |
| RI | mean | 0·7031 | 0·6834 | 0·4098 | 0·5887 |
|  | SD | 0·1382 | 0·1547 | 0·2142 | 0·1517 |
|  | median | 0·7310 | 0·7150 | 0·4090 | 0·6010 |
|  | IQR | [0·6410, 0·8010] | [0·5780, 0·7910] | [0·2405, 0·5615] | [0·4755, 0·6980] |
| Rest Amount | mean | 8·3 | 8·9 | 7·8 | 9·1 |
|  | SD | 1·5 | 1·5 | 2·0 | 2·1 |
|  | median | 9·3 | 9·0 | 8·1 | 9·1 |
|  | IQR | [7·0, 9·4] | [8·2, 9·6] | [6·4, 9·1] | [8·0, 10·4] |
| Centre time | mean | 3·1 | 4·0 | 10·8 | 5·0 |
|  | SD | 1·2 | 1·4 | 1·9 | 1·9 |
|  | median | 3·1 | 3·8 | 10·6 | 4·6 |
|  | IQR | [2·3, 3·8] | [3·4, 4·7] | [9·4, 11·8] | [3·4, 6·6] |

**Supplementary Table S2c. P-values of two-sample t-test between DS and NS, and between work and free days.**

|  |  | p1-1 | RI | Rest Amount | Centre Time of rest |
| --- | --- | --- | --- | --- | --- |
| DS vs NS | Workdays | <0·0001 | <0·0001 | 0·14 | <0·0001 |
|  | Free days | 0·010 | 0·0005 | 0·42 | 0·0013 |
| Work vs Free days | DS | 0·32 | 0·26 | 0·0010 | <0·0001 |
|  | NS | 0·21 | <0·0001 | <0·0001 | <0·0001 |

Circadian parameters between DS and NS were compared via Mann-Whitney U Test. Circadian parameters between work and free days were compared via Wilcoxon Signed Rank Test.

**Supplementary** **Table S3a. Comparison of 3 DS clusters.**

|  | |  | Cluster 1 (n=34) | | Cluster 2 (n=24) | | Cluster 3 (n=19) | | p-value | |
| --- | --- | --- | --- | --- | --- | --- | --- | --- | --- | --- |
| p1-1 | | Workdays (mean sd) | 0·9478 | 0·0238 | 0·9313 | 0·0457 | 0·9568 | 0·0158 | 0·081 |  |
|  | | Free days | 0·9321 | 0·0297 | 0·9462 | 0·0398 | 0·9542 | 0·0348 | 0·0039 | ** |
| RI | | Workdays | 0·7418 | 0·1221 | 0·6674 | 0·1544 | 0·6791 | 0·1329 | 0·11 |  |
|  | | Free days | 0·6240 | 0·1368 | 0·7402 | 0·1623 | 0·7178 | 0·1447 | 0·0086 | ** |
| Rest amount | | Workdays | 9·3 | 1·2 | 6·8 | 0·8 | 8·3 | 1·4 | <0·0001 | *** |
|  | | Free days | 8·9 | 1·7 | 9·0 | 1·0 | 8·9 | 1·6 | 0·53 |  |
| Rest centre time | | Workdays | 3·3 | 1·0 | 2·3 | 0·9 | 3·9 | 1·3 | <0·0001 | *** |
|  | | Free days | 3·1 | 1·1 | 4·0 | 0·6 | 5·7 | 1·1 | <0·0001 | *** |
|  | |  |  |  |  |  |  |  |  |  |
| Age | |  | 38·1 | 8·8 | 39·2 | 13·0 | 34·3 | 11·0 | 0·30 |  |
| BMI | |  | 26·2 | 5·7 | 25·0 | 6·1 | 24·3 | 3·9 | 0·39 |  |
| Chronotype score | |  | 53·8 | 7·9 | 56·0 | 7·3 | 49·3 | 9·0 | 0·062 | . |
| Cohabitant | | No  Yes | 8  26 | 24%  76% | 7  17 | 29%  71% | 11  8 | 58%  42% | 0·034 | * |
| Day shift type | | Morning  Mixed  Afternoon | 6  17  11 | 18%  50%  32% | 11  12  1 | 46%  50%  4% | 0  16  3 | 0%  84%  16% | 0·0007 | *** |
| Menopause | post-menopause  pre-menopause | | 4  29 | 12%  88% | 7  16 | 30%  70% | 2  16 | 11%  89% | 0·15 |  |
| Sleep debt | |  | 0·9 | 1·1 | 1·2 | 1·7 | 1·1 | 1·6 | 0·44 |  |
| Sleep duration (Q) | | Workdays | 6·6 | 1·1 | 6·8 | 1·6 | 6·9 | 1·4 | 0·69 |  |
|  | | Free days | 8·1 | 1·5 | 8·8 | 1·4 | 9·0 | 1·3 | 0·11 |  |
| Sleep duration (D) | | Workdays | 7·1 | 1·2 | 6·1 | 1·1 | 6·7 | 1·3 | 0·012 | * |
|  | | Free days | 8·1 | 1·3 | 8·3 | 1·0 | 8·1 | 1·2 | 0·89 |  |
| Dominant period (Chesttemp) | | 24 h  12 h  8 h  Others | 28  1  1  4 | 82%  3%  3%  12% | 13  7  1  3 | 54%  29%  4%  13% | 13  4  0  2 | 68%  21%  0%  11% | 0·17 |  |
|  | | mean | 25·3 | 8·0 | 21·8 | 9·4 | 20·6 | 5·0 | 0·046 | * |

Sleep duration (Q) denotes the nocturnal sleep duration from questionnaire. Sleep duration (D) denotes the sleep duration per 24h from diary. Menopause is considered only for female subjects and every cluster contains one male. Cohabitant and categorical dominant period were compared via Chi-square test. Others were compared via Kruskal-Wallis test. Significance codes: 0 ‘***’ 0·001 ‘**’ 0·01 ‘*’ 0·05 ‘.’ 0·1 ‘ ’ 1

**Supplementary** **Table S3b. Comparison of 3 NS clusters.**

|  | |  | | Cluster 1 (n=28) | | Cluster 2 (n=11) | | Cluster 3 (n=24) | | p-value | |
| --- | --- | --- | --- | --- | --- | --- | --- | --- | --- | --- | --- |
| p1-1 | | Workdays (mean sd) | | 0·9236 | 0·0385 | 0·9176 | 0·0563 | 0·9211 | 0·0540 | 0·96 |  |
|  | | Free days | | 0·9251 | 0·0554 | 0·9264 | 0·0411 | 0·9184 | 0·0641 | 0·67 |  |
| RI | | Workdays | | 0·3964 | 0·2117 | 0·2655 | 0·1418 | 0·4917 | 0·2124 | 0·011 | * |
|  | | Free days | | 0·6565 | 0·1428 | 0·4552 | 0·0948 | 0·5707 | 0·1405 | 0·0090 | ** |
| Rest amount | | Workdays | | 7·4 | 1·8 | 8·0 | 2·3 | 8·2 | 2·0 | 0·33 |  |
|  | | Free days | | 8·8 | 1·8 | 8·7 | 2·1 | 9·7 | 2·3 | 0·17 |  |
| Rest centre time | | Workdays | | 11·1 | 1·7 | 8·8 | 1·4 | 11·3 | 1·7 | <0·0001 | *** |
|  | | Free days | | 3·5 | 0·9 | 3·9 | 1·2 | 7·1 | 0·8 | <0·0001 | *** |
|  | |  | |  |  |  |  |  |  |  |  |
| Age | |  | | 42·8 | 9·0 | 49·4 | 10·0 | 43·2 | 11·7 | 0·13 |  |
| BMI | |  | | 25·3 | 3·9 | 28·6 | 4·4 | 26·8 | 5·5 | 0·096 | . |
| Chronotype score | |  | | 51·4 | 9·2 | 50·5 | 11·4 | 41·3 | 8·5 | 0·0004 | *** |
| Cohabitant | | No  Yes | | 7  21 | 25%  75% | 3  8 | 27%  73% | 13  11 | 54%  46% | 0·073 | . |
| Menopause | post-menopause  pre-menopause | | | 10  16 | 38%  62% | 5  5 | 50%  50% | 11  12 | 48%  52% | 0·74 |  |
| # of years in night shift work | | |  | 8·7 | 7·8 | 16·0 | 11·8 | 7·7 | 8·1 | 0·020 | * |
| Sleep debt | |  | | 2·3 | 1·6 | 2·3 | 1·7 | 2·0 | 1·6 | 0·69 |  |
| Sleep duration (Q) | | Workdays | | 5·0 | 1·8 | 4·1 | 2·1 | 5·5 | 1·4 | 0·094 | . |
|  | | Free days | | 8·5 | 1·4 | 7·1 | 2·3 | 8·6 | 1·8 | 0·058 | . |
| Sleep duration (D) | | Workdays | | 5·2 | 1·2 | 4·7 | 2·1 | 5·4 | 1·4 | 0·31 |  |
|  | | Free days | | 8·2 | 1·2 | 8·3 | 2·2 | 9·1 | 1·4 | 0·050 | . |
| Dominant period (Chesttemp) | | 24 h  12 h  8 h  Others | | 13  8  2  5 | 46%  29%  7%  18% | 7  3  0  1 | 64%  27%  0%  9% | 10  8  4  2 | 42%  33%  17%  8% | 0·61 |  |
|  | | mean | | 20·8 | 8·3 | 21·9 | 6·2 | 17·5 | 7·3 | 0·15 |  |

Sleep duration (Q) denotes the nocturnal sleep duration from questionnaire. Sleep duration (D) denotes the sleep duration per 24h from diary. Menopause is considered only for female subjects. There are 2, 1 and 1 male in cluster 1, 2 and 3, respectively. Cohabitant and categorical dominant period were compared via Chi-square test. Others were compared via Kruskal-Wallis test. Significance codes: 0 ‘***’ 0·001 ‘**’ 0·01 ‘*’ 0·05 ‘.’ 0·1 ‘ ’ 1

**Supplementary** **Table S4a. Pairwise comparison of 3 DS clusters.**

|  |  | | Mean | | | p-value | | | |
| --- | --- | --- | --- | --- | --- | --- | --- | --- | --- |
|  |  | | Cluster 1 (n=34) | Cluster 2 (n=24) | Cluster 3 (n=19) | | 1 vs 2 | 1 vs 3 | 2 vs 3 |
| p1-1 | Workdays | | 0·9478 | 0·9313 | 0·9568 | | 0·39 | 0·39 | 0·075 |
|  | Free days | | 0·9321 | 0·9462 | 0·9542 | | 0·042 | 0·0057 | 0·38 |
| RI | Workdays | | 0·7418 | 0·6674 | 0·6791 | | 0·19 | 0·22 | 0·90 |
|  | Free days | | 0·6240 | 0·7402 | 0·7178 | | 0·015 | 0·048 | 0·74 |
| Rest amount | Workdays | | 9·3 | 6·8 | 8·3 | | <0·0001 | 0·035 | 0·0021 |
|  | Free days | | 8·9 | 9·0 | 8·9 | | 0·89 | 0·87 | 0·89 |
| Rest centre time | Workdays | | 3·3 | 2·3 | 3·9 | | 0·0012 | 0·18 | <0·0001 |
|  | Free days | | 3·1 | 4·0 | 5·7 | | 0·013 | <0·0001 | 0·0002 |
|  |  | |  |  |  | |  |  |  |
| Age |  | | 38·1 | 39·2 | 34·3 | | 0·99 | 0·46 | 0·45 |
| BMI |  | | 26·2 | 25·0 | 24·3 | | 0·64 | 0·64 | 0·90 |
| Chronotype score |  | | 53·8 | 55·9 | 49·3 | | 0·37 | 0·19 | 0·058 |
| Sleep debt |  | | 0·9 | 1·2 | 1·1 | | 0·80 | 0·80 | 0·88 |
| Sleep duration (Q) | Workdays | | 6·6 | 6·8 | 6·9 | | 1·00 | 1·00 | 1·00 |
|  | Free days | | 8·1 | 8·8 | 9·0 | | 0·22 | 0·19 | 0·73 |
| Sleep duration (D) | Workdays | | 7·1 | 6·1 | 6·7 | | 0·010 | 0·51 | 0·11 |
|  | Free days | | 8·1 | 8·3 | 8·1 | | 1·00 | 1·00 | 1·00 |
| Dominant period (Chesttemp) | |  | 25·3 | 21·8 | 20·6 | | 0·41 | 0·039 | 0·22 |

Comparison among clusters were conducted via Dunn’s non-parametric all-pairs comparison test with Holm method for p-value adjustment.

**Supplementary Table S4b. Pairwise comparison of 3 NS clusters.**

|  |  | | | Mean | | | p-value | | | |
| --- | --- | --- | --- | --- | --- | --- | --- | --- | --- | --- |
|  |  | | | Cluster 1 (n=34) | Cluster 2 (n=24) | Cluster 3 (n=19) | | 1 vs 2 | 1 vs 3 | 2 vs 3 |
| p1-1 | Workdays | | | 0·9236 | 0·9176 | 0·9211 | | 1·00 | 1·00 | 1·00 |
|  | Free days | | | 0·9251 | 0·9264 | 0·9184 | | 1·00 | 1·00 | 1·00 |
| RI | Workdays | | | 0·3964 | 0·2655 | 0·4917 | | 0·14 | 0·14 | 0·0088 |
|  | Free days | | | 0·6565 | 0·4552 | 0·5707 | | 0·0008 | 0·078 | 0·078 |
| Rest amount | Workdays | | | 7·4 | 8·0 | 8·2 | | 0·82 | 0·44 | 0·82 |
|  | Free days | | | 8·8 | 8·7 | 9·7 | | 0·56 | 0·29 | 0·27 |
| Rest centre time | Workdays | | | 11·1 | 8·8 | 11·3 | | 0·0010 | 0·72 | 0·0007 |
|  | Free days | | | 3·5 | 3·9 | 7·1 | | 0·50 | <0·0001 | 0·0001 |
|  |  | | |  |  |  | |  |  |  |
| Age |  | | | 42·8 | 49·2 | 43·2 | | 0·14 | 0·75 | 0·18 |
| BMI |  | | | 25·3 | 28·6 | 26·8 | | 0·094 | 0·34 | 0·34 |
| Chronotype score |  | | | 51·5 | 50·5 | 41·3 | | 0·80 | 0·0006 | 0·018 |
| # of years in night shift work | |  | | 8·7 | 16·0 | 7·7 | | 0·18 | 0·49 | 0·082 |
| Sleep debt |  | | | 2·3 | 2·3 | 2·0 | | 1·00 | 1·00 | 1·00 |
| Sleep duration (Q) | Workdays | | | 5·0 | 4·1 | 5·5 | | 0·41 | 0·40 | 0·095 |
|  | Free days | | | 8·5 | 7·1 | 8·6 | | 0·080 | 0·91 | 0·080 |
| Sleep duration (D) | Workdays | | | 5·2 | 4·7 | 5·4 | | 0·43 | 0·71 | 0·39 |
|  | Free days | | | 8·2 | 8·3 | 9·1 | | 0·53 | 0·046 | 0·41 |
| Dominant period (Chesttemp) | | |  | 20·8 | 21·9 | 17·5 | | 0·39 | 0·39 | 0·18 |

Comparison among clusters were conducted via Dunn’s non-parametric all-pairs comparison test with Holm method for p-value adjustment.

**Supplementary Table S5. Examples of copula function with range of corresponding parameter** $\boldsymbol{\theta}$ **and number of degrees of freedom** $\boldsymbol{\zeta}$ **when present, and relation between Kendall’s** $\boldsymbol{\tau}$ **correlation coefficient and** $\boldsymbol{\theta}$**.**

| Copula | $\boldsymbol{C(u,v; \zeta, \theta)}$ | Ranges of $\boldsymbol{\theta}$ | Kendall’s $\boldsymbol{\tau}$ |
| --- | --- | --- | --- |
| AMH | $\frac{uv}{1-\theta(1-u)(1-v)}$ | $\theta\epsilon[-1, 1]$ | $-\frac{2}{3\theta^{2}}\left[ \theta+\left( 1-\theta\right)^{2}\log\left( 1-\theta\right) \right]+1$ |
| Frank | $-\theta^{-1}log\left[ 1+\left( e^{-\theta u}-1 \right)\left( e^{-\theta v}-1 \right)/\left( e^{-\theta}-1 \right) \right]$ | $\theta\epsilon\mathbb{R \backslash} \left\{ 0 \right\}$ | $1-\frac{4}{\theta}\left[ 1-D_{1}\left( \theta\right) \right]$ |
| Gaussian | $\Phi_{2}\left( \Phi^{-1}\left( u \right),\Phi^{-1}\left( v \right); \theta\right)$ | $\theta\epsilon[-1, 1]$ | $\frac{2}{\pi}arcsin\left( \theta\right)$ |

The $D_{1}\left( \theta\right)=\frac{1}{\theta} \int_{0}^{\theta} \frac{1}{exp\left( t \right)-1}dt$ is the Debye function. The CDF of a standard bivariate normal distribution with correlation coefficient $\theta$ and a univariate standard normal distribution is denoted by $\Phi_{2}\left( \cdot, \cdot;\theta\right)$ and $\Phi(\cdot)$ respectively. Note that $\zeta$ is only needed for the Student-t copula function which is not included in this table because it was not used in the models. The range of Kendall’s $\tau$ is (-0·18, 0·33), (-1, 1)$\backslash\left\{ 0 \right\}$, and [-1, 1] for AMH, Frank and Gaussian copula respectively· Higher $\theta$ results in higher $\tau$. The AMH copula function was used in the regression for log odds of p1-1; the Frank one was used for log odds of RI and rest centre time; the Gaussian one was used for rest amount.

**Supplementary Table S6a.** **TRIPOD Checklist**

| **Section/Topic Item Checklist Item Section*** | | | |
| --- | --- | --- | --- |
| **Title and abstract** | | | |
| Title | 1 | Identify the study as developing and/or validating a multivariable prediction model, the target population, and the outcome to be predicted. | i |
| Abstract | 2 | Provide a summary of objectives, study design, setting, participants, sample size, predictors, outcome, statistical analysis, results, and conclusions. | ii |
| **Introduction** | | | |
| Background and objectives | 3a | Explain the medical context (including whether diagnostic or prognostic) and rationale for developing or validating the multivariable prediction model, including references to existing models. | 1 |
|  | 3b | Specify the objectives, including whether the study describes the development or validation of the model or both. | 1 |
| **Methods** | | | |
| Source of data | 4a | Describe the study design or source of data (e.g., randomized trial, cohort, or  registry data), separately for the development and validation data sets, if applicable. | 2.2 |
|  | 4b | Specify the key study dates, including start of accrual; end of accrual; and, if applicable, end of follow-up. | Figure 1a |
| Participants | 5a | Specify key elements of the study setting (e.g., primary care, secondary care, general population) including number and location of centres. | 2.1 |
|  | 5b | Describe eligibility criteria for participants. | 2.2,  Figure 1a |
|  | 5c | Give details of treatments received, if relevant. |  |
| Outcome | 6a | Clearly define the outcome that is predicted by the prediction model, including how and when assessed. | 2.3 |
|  | 6b | Report any actions to blind assessment of the outcome to be predicted. |  |
| Predictors | 7a | Clearly define all predictors used in developing or validating the multivariable  prediction model, including how and when they were measured. | 2.3 |
|  | 7b | Report any actions to blind assessment of predictors for the outcome and other predictors. |  |
| Sample size | 8 | Explain how the study size was arrived at. | 2.3 |
| Missing data | 9 | Describe how missing data were handled (e.g., complete-case analysis, single imputation, multiple imputation) with details of any imputation method. |  |
| Statistical analysis methods | 10a | Describe how predictors were handled in the analyses. | 2.3 |
|  | 10b | Specify type of model, all model-building procedures (including any predictor selection), and method for internal validation. | 2.3 |
|  | 10d | Specify all measures used to assess model performance and, if relevant, to compare multiple models. |  |
| Risk groups | 11 | Provide details on how risk groups were created, if done. |  |
| **Results** | | | |
| Participants | 13a | Describe the flow of participants through the study, including the number of participants with and without the outcome and, if applicable, a summary of the follow-up time. A diagram may be helpful. | 3.1 |
|  | 13b | Describe the characteristics of the participants (basic demographics, clinical features, available predictors), including the number of participants with missing  data for predictors and outcome. | 3.1 |
| Model development | 14a | Specify the number of participants and outcome events in each analysis. | 3.1 |
|  | 14b | If done, report the unadjusted association between each candidate predictor and outcome. |  |
| Model specification | 15a | Present the full prediction model to allow predictions for individuals (i.e., all regression coefficients, and model intercept or baseline survival at a given time  point). | 3 |
|  | 15b | Explain how to the use the prediction model. |  |
| Model performance | 16 | Report performance measures (with CIs) for the prediction model. |  |
| **Discussion** | | | |
| Limitations | 18 | Discuss any limitations of the study (such as nonrepresentative sample, few events  per predictor, missing data). | 4 |
| Interpretation | 19b | Give an overall interpretation of the results, considering objectives, limitations, and  results from similar studies, and other relevant evidence. | 4 |
| Implications | 20 | Discuss the potential clinical use of the model and implications for future research. | 4 |
| **Other information** | | | |
| Supplementary information | 21 | Provide information about the availability of supplementary resources, such as study protocol, Web calculator, and data sets. |  |
| Funding | 22 | Give the source of funding and the role of the funders for the present study. | 2.4, 7 |

*See Table S6b for the section name and index.

**Supplementary Table S6b. Section name and index**

| **Section name** | **Section index** |
| --- | --- |
| Title | i |
| Abstract | ii |
| Research in context | iii |
| Introduction | 1 |
| Method | 2 |
| Ethic | 2.1 |
| Study design and participants | 2.2 |
| Statistics | 2.3 |
| Role of funders | 2.4 |
| Results | 3 |
| Participants’ Characteristics and Study Conduct | 3.1 |
| Circadian parameters and chest temperature over the entire study session | 3.2 |
| Circadian parameters on work versus free days | 3.3 |
| Clustering of individual daily rest profiles | 3.4 |
| Predictors of circadian parameters | 3.5 |
| Ultradian oscillations in PA and Chesttemp during rest | 3.6 |
| Discussion | 4 |
| Contributors | 5 |
| Declaration of Interests | 6 |
| Acknowledgments | 7 |
| Code and Data Sharing Statement | 8 |

Supplementary References

1. Horne JA, Östberg O. A self-assessment questionnaire to determine morningness-eveningness in human circadian rhythms. Vol. 4, International Journal of Chronobiology. Östberg, O.: Department of Human Work Sciences, University of Lulea, Lulea, Sweden, S-95187: Gordon and Breach Science Pub Ltd; 1976. p. 97–110.

2. Taillard J, Philip P, Chastang J-F, Bioulac B. Validation of Horne and Ostberg morningness-eveningness questionnaire in a middle-aged population of French workers. J Biol Rhythms. 2004;19(1):76–86.

3. Leger D, Richard JB, Collin O, Sauvet F, Faraut B. Napping and weekend catchup sleep do not fully compensate for high rates of sleep debt and short sleep at a population level (in a representative nationwide sample of 12,637 adults). Sleep Med. 2020;74:278–88.

4. Huang Q, Cohen D, Komarzynski S, Li X-M, Innominato P, Lévi F, et al. Hidden Markov models for monitoring circadian rhythmicity in telemetric activity data. J R Soc Interface. 2018 Feb 1;15(139):20170885.

5. Zucchini W, Macdonald IL, Langrock R. Hidden Markov models for time series: An introduction using R, second edition. Chapman and Hall/CRC. CRC Press; 2017. 1–370 p.

6. Huang Q, Cohen D, Komarzynski S, Li XM, Innominato P, Lévi F, et al. Hidden Markov models for monitoring circadian rhythmicity in telemetric activity data. J R Soc Interface. 2018;15(139).

7. Banachewicz K, Lucas A, Van Der Vaart A. Modelling portfolio defaults using hidden markov models with covariates. Econom J. 2008;11(1):155–71.

8. Ward JH. Hierarchical Grouping to Optimize an Objective Function. J Am Stat Assoc. 1963;58(301):236–44.

9. Thorndike RL. Who belongs in the family? Psychometrika. 1953;18(4):267–76.

10. Marra G, Radice R. Bivariate copula additive models for location, scale and shape. Comput Stat Data Anal. 2017;112:99–113.

11. Sklar M. Fonctions de repartition a n dimensions et leurs marges. Publ Inst Stat Univ Paris. 1959;8:229–31.

12. Trivedi PK, Zimmer DM. Copula Modeling: An Introduction for Practitioners. Foundations and Trends(R) in Econometrics. 2005. p. 1–111.

13. Hurvich CM, Tsai CL. Regression and time series model selection in small samples. Biometrika. 1989;76(2):297–307.

14. Hocking RR. A Biometrics Invited Paper. The Analysis and Selection of Variables in Linear Regression. Biometrics. 1976;32(1):1.

15. Cook RD. Detection of Influential Observation in Linear Regression. Technometrics. 1977;19(1):15–8.
